# Target highlights in CASP13: Experimental target structures through the eyes of their authors

**DOI:** 10.1002/prot.25805

**Published:** 2019-09-09

**Authors:** Rosalba Lepore, Andriy Kryshtafovych, Markus Alahuhta, Harshul A. Veraszto, Yannick J. Bomble, Joshua C. Bufton, Alex N. Bullock, Cody Caba, Hongnan Cao, Owen R. Davies, Ambroise Desfosses, Matthew Dunne, Krzysztof Fidelis, Celia W. Goulding, Manickam Gurusaran, Irina Gutsche, Christopher J. Harding, Marcus D. Hartmann, Christopher S. Hayes, Andrzej Joachimiak, Petr G. Leiman, Peter Loppnau, Andrew L. Lovering, Vladimir V. Lunin, Karolina Michalska, Ignacio Mir‐Sanchis, AK Mitra, John Moult, George N. Phillips Jr, Daniel M. Pinkas, Phoebe A. Rice, Yufeng Tong, Maya Topf, Jonathan D. Walton, Torsten Schwede

**Affiliations:** ^1^ BSC‐CNS Barcelona Supercomputing Center Barcelona Spain; ^2^ Genome Center University of California Davis California; ^3^ Biosciences Center National Renewable Energy Laboratory Golden Colorado; ^4^ Department of Protein Evolution Max Planck Institute for Developmental Biology Tübingen Germany; ^5^ Nuffield Department of Medicine; Structural Genomics Consortium University of Oxford Oxford UK; ^6^ School of Biochemistry University of Bristol Bristol UK; ^7^ Department of Chemistry and Biochemistry University of Windsor Windsor Ontario Canada; ^8^ Department of BioSciences Rice University Houston Texas; ^9^ Great Lakes Bioenergy Research Center University of Wisconsin Madison Wisconsin; ^10^ Institute for Cell and Molecular Biosciences, Faculty of Medical Sciences Newcastle University Newcastle upon Tyne UK; ^11^ School of Biological Sciences University of Auckland Auckland New Zealand; ^12^ Institut de Biologie Structurale Université Grenoble Alpes, CEA, CNRS Grenoble France; ^13^ Institute of Food, Nutrition and Health Zurich Switzerland; ^14^ Department of Molecular Biology and Biochemistry; Pharmaceutical Sciences University of California Irvine Irvine California; ^15^ School of Biosciences University of Birmingham Birmingham UK; ^16^ Department of Molecular, Cellular and Developmental Biology, Biomolecular Science and Engineering Program University of California Santa Barbara California; ^17^ Structural Biology Center, Biosciences Division Midwest Center for Structural Genomics Argonne; ^18^ Department of Biochemistry and Molecular Biology University of Chicago Chicago Illinois; ^19^ Department of Biochemistry and Molecular Biology, Sealy Center for Structural Biology and Molecular Biophysics University of Texas Medical Branch Galveston Texas; ^20^ Structural Genomics Consortium University of Toronto Toronto Ontario Canada; ^21^ Department of Biochemistry and Molecular Biology The University of Chicago Chicago Illinois; ^22^ Institute for Bioscience and Biotechnology Research, Department of Cell Biology and Molecular genetics University of Maryland Rockville Maryland USA; ^23^ Institute of Structural and Molecular Biology, Birkbeck University College London London UK; ^24^ Great Lakes Bioenergy Research Center and Department of Plant Biology Michigan State University East Lansing Michigan; ^25^ Biozentrum University of Basel Basel Switzerland; ^26^ SIB Swiss Institute of Bioinformatics Biozentrum University of Basel Basel Switzerland

**Keywords:** CASP, protein structure prediction, cryo‐EM, X‐ray crystallography

## Abstract

The functional and biological significance of selected CASP13 targets are described by the authors of the structures. The structural biologists discuss the most interesting structural features of the target proteins and assess whether these features were correctly reproduced in the predictions submitted to the CASP13 experiment.

**Abbreviations**
CASP: community wide experiment on the Critical Assessment of Techniques for Protein Structure Prediction
DDR: DNA damage repair
Gal: d‐galactose
Glc: d‐glucose
PPI: protein‐protein interaction
RFWD3: RING finger and WD repeat domain‐containing protein 3
WDR: WD40‐repeat
Xyl: d‐xylose

## 1 INTRODUCTION

Community wide experiment on the Critical Assessment of Techniques for Protein Structure Prediction (CASP) operation would not be possible without the help of experimental structural biologists, who agree to share with the CASP organization their work‐in‐progress or recently solved protein structures in advance of their public release. In the latest round of CASP (CASP13, 2018), 75 proteins and protein complexes were suggested as modeling targets by 36 structure determination groups from 14 countries. All suggested entries were released for prediction, however, eight of them were canceled due to lack of structure by the time of the assessment or release of relevant structural information before the end of the target prediction season. Of the remaining 67 entries, 58 were solved by X‐ray crystallography, 7 with cryo‐EM and 2 by NMR. When classified by quaternary structure, 25 entries were monomeric, 30 homo‐oligomeric and 12 heteromeric. The heteromeric complexes were released for prediction (and later assessed) as both, whole multimolecular complexes (12) and constitutive subunits (25). All in all, 80 single‐molecule targets and 42 multi‐molecule targets were part of the CASP13 experiment. CASP organizers, who are co‐authors of this article, want to thank every experimentalist who contributed to CASP13 (see Table S1) and this way helped developing more effective protein structure prediction methods.

The chapters of the article reflect the views of the contributing authors on 13 CASP13 targets (Table 1), including three monomeric: the *Arabidopsis thaliana* xylan *O*‐acetyltransferase 1 (T0969), the LP1413 single‐strand DNA binding protein (T0958), the WD40‐repeat domain of the human E3 ubiquitin ligase RFWD3 (T0954); 4 homo‐oligomeric: the H1 domain of human KCTD8 (T0970), a putative ACAD from *B. bacteriovorus* (T0961), a glycoside hydrolase family 31 α‐xylosidase (T1009), the pentafunctional AROM Complex from *Chaetomium thermophilum* (T0999); 6 hetero‐oligomeric: the receptor‐binding tip (gp37_3_‐gp38) from the *Salmonella* phage S16 (H0953), the toxin‐immunity protein complex from *Escherichia coli* 3006 (H0957), and *Klebsiella pneumoniae* 342 (H0968), the human MAJIN‐TERB2 hetero‐tetrameric complex (H0980), the apical end cap of the antifeeding prophage (AFP) from *Serratia entomophila* and its threefold symmetric needle (H1021 and H1022).

**Table 1:** CASP13 target highlights

						CASP13 assessment		
Target	PDB	Method	Resolution (å)	Stoichiometry	Size	Protomer		Assembly
						GDT‐TS	IDDT	QS‐score
T0969	6CCI	X‐ray	1.85	A1	487	58.19	0.51	‐
T0958	6BTC	X‐ray	2.18	A1	96	80.84	0.69	‐
T0954	6CVZ	X‐ray	1.80	A1	350	72.02	0.66	‐
T0970	6G57	X‐ray	2.80	A2	97	67.94	0.28	0.64
T0961	6SD8	X‐ray	1.50	A4	505	91.65	0.80	0.91
T1009	6DRU	X‐ray	2.70	A2	718	71.24	0.64	0.18
T0999	N/A	X‐ray	3.00	A2	1589	80.39	0.73	0.82
H0953	6F45	X‐ray	1.70	A3B1	72 249	54.48 40.12	0.63 0.40	0.37
H0957	6CP8	X‐ray	2.20	A1B1	163 164	45.22 60.97	0.57 0.57	0.07
H0968	6CP9	X‐ray	2.55	A2B2	126 116	71.40 78.70	0.61 0.66	0.14
H0980	6GNX	X‐ray	2.90	A2B2	111 52	54.81 ‐	0.45 ‐	0.08
H1021	6RAP	EM	3.30	A6B6C6	149 354 295	75.67 68.70 36.77	0.65 0.58 0.57	0.33
H1022	6RBK	EM	3.40	A6B3	229 529	43.61 62.25	0.55 0.59	0.43

*Note*: Columns indicate target ID, PDB ID, experimental method, resolution, stoichiometry, size, and CASP13 assessment results. For each target, the accuracy of the best model 1 is provided both at the level of individual protomers (best GDT‐TS and corresponding IDDT score) and full assembly (best QS‐score).

The results of the comprehensive numerical evaluation of CASP13 models are available at the Prediction Center website (http://www.predictioncenter.org). The detailed assessment of the models by the assessors is provided elsewhere in this issue.

## 2 RESULTS

### 2.1 Structure of the WD40‐repeat domain of the human E3 ubiquitin ligase RFWD3 (CASP: T0954; PDB: 6CVZ). Provided by Cody Caba, Peter Loppnau, and Yufeng Tong

Ubiquitination is the covalent attachment of ubiquitin (Ub) to a substrate lysine. It represents the second most prevalent post‐translational modification and follows a three‐step enzymatic cascade involving an E1 Ub‐activating enzyme, E2 Ub‐conjugating enzyme, and an E3 Ub‐protein ligase. The diverse distribution of E3s, for which >600 are known to exist in the human genome, affords the ubiquitome pervasive substrate specificity. As such, Ub‐modified targets are ultimately destined for a myriad of molecular outcomes depending on the Ub chain length and linkage type.^1^ Many proteins of the E3 superfamily incorporate the highly abundant WD40‐repeat (WDR) substrate‐recruiting domain as a functional protein‐nucleic acid and protein‐protein interaction (PPI) module. As a vital component to many multiprotein complexes, the WDR domain is unsurprisingly central to a range of cellular processes, including checkpoint signaling, protein trafficking and degradation, DNA replication, and DNA damage repair (DDR). RFWD3 (RING finger and WD repeat domain‐containing protein 3) is a WDR‐containing E3 originally identified as an ATM/ATR substrate involved in DDR.^2^, ^3^ Evidence has shown that the WDR domain is primarily responsible for the functional interactions that allow RFWD3 to maintain genomic stability.^4^, ^5^ Furthermore, heritable mutations, particularly an Ile639Lys point mutation within the WDR domain, may lead to the rare genomic instability disease known as Fanconi anemia (FA),^6^ thereby implicating *RFWD3* as a potential FA‐associated gene (alias: *FANCW*).^4^, ^5^, ^6^ Until recently, the biochemical characterization of RFWD3 has lacked complementary high‐resolution structural information. Here, we discuss the 1.8 å resolution X‐ray crystal structure of the C‐terminal WDR domain of human RFWD3 (Table 1, Target: T0954; PDB: 6CVZ).

**Figure 1:**
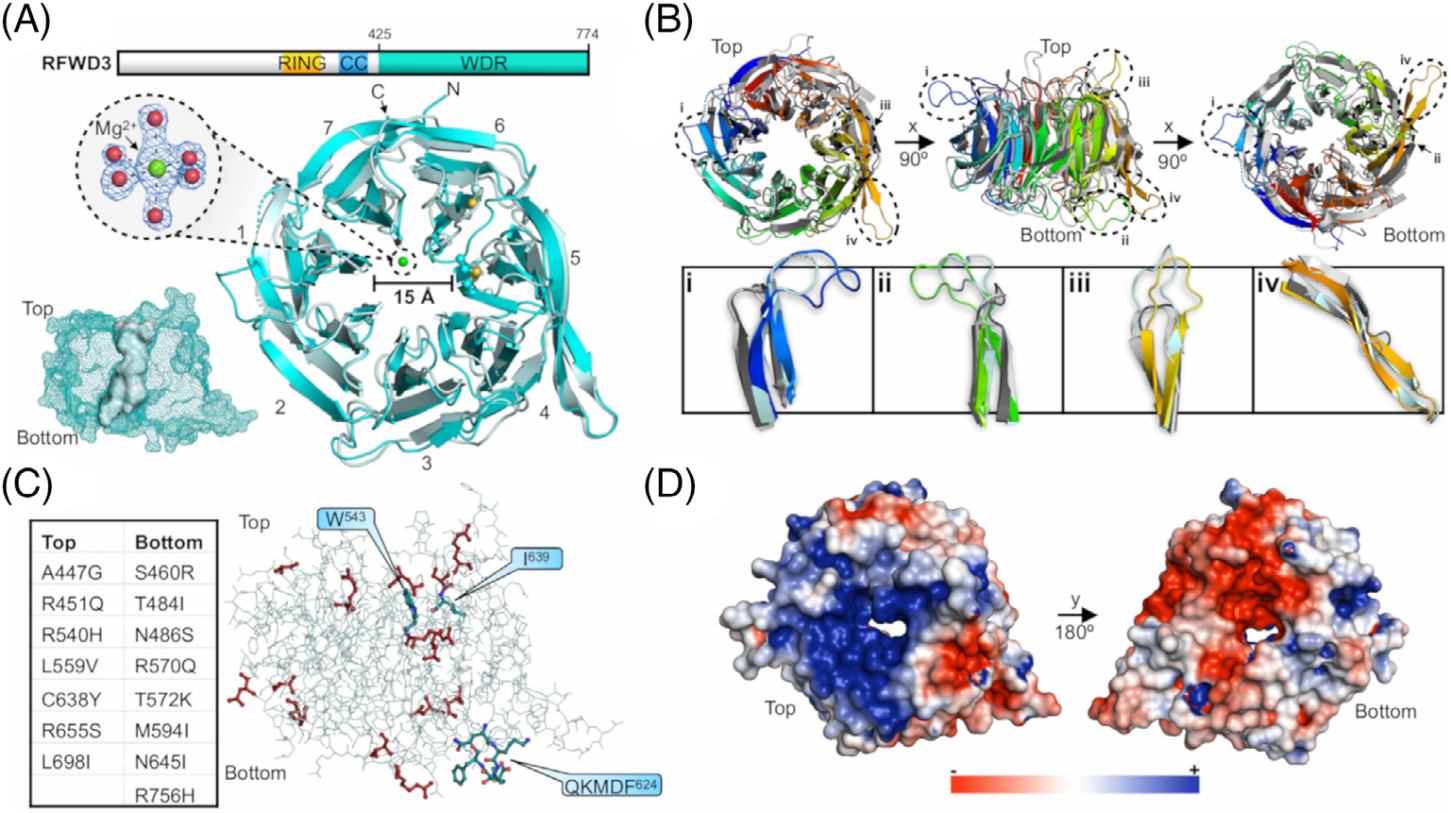
The WDR domain of human RFWD3. A, Schematic representation of RFWD3's domain organization and cartoon representation of the WDR domain crystal structure (6CVZ; cyan) aligned with the most accurately predicted model T0954TS043_1 (light blue). All seven blades of the β‐propeller architecture are labeled. The magnesium ion (green sphere) is octahedrally coordinated by the oxygen of six water molecules (oxygen atoms shown as red spheres). The *2mF*_*o*_ *‐DF*_*c*_ electron density map is shown contoured to 2.0 *σ*. The volume of the central cavity is provided as a surface representation (determined using POCASA v. 1.1). RING, really interesting new gene; CC, coiled‐coil. B, Alignment of 6CVZ (rainbow), 6DAS (dark gray), 5HQG (light gray), and model T0954TS043_1 (light blue). Loops of interest are circled and labeled i to iv, respectively. C, Visualization of the key residues involved in DDR (blue) and the solvent‐exposed residues identified to be mutated in various cancers using the COSMIC database (red). The disease‐related point mutations (red) are tabulated. D, Electrostatic potential map of 6CVZ from −3 kT/e (red) to +3 kT/e (blue). All structural figures were prepared using PyMOL (v. 2.3.0, Schrödinger LLC)

WDR domains exhibit a β‐propeller architecture typically composed of seven WD repeats (propeller blades). Each repeat is a four‐stranded antiparallel β‐sheet of 40 to 60 residues in length. Other features, though not conserved, may exist within the repeats, such as a DH(S/T)W hydrogen‐bonded tetrad, or GH and WD dipeptides. Due to low sequence homology, predicting the presence of WDR domains with sequence analysis alone is difficult and results in an underrepresentation within the proteome.^7^ Some of the common protein sequence analysis databases predict RFWD3 to contain three distinct WDRs, while the more specialized WDSP database^7^ suggests the presence of six^8^; however, our structure reveals seven. Stabilizing the fold are hydrophobic interactions between adjacent repeats, along with a velcro closure between the first and last repeats. Additionally, there is an exposed disulfide bond on the top surface linking repeats five and six (Cys638 to Cys696, Figure 1A). It is yet to be determined whether this is important for conformational stability or a potential site of redox‐regulated activity. Multiple sequence alignment suggests these cysteine residues are not well conserved. Additionally, only a single WD dipeptide is present (WD^730^), located at the C‐terminus of the third strand in repeat six. It should also be noted that a coordinated magnesium ion in the structural model is an artifact of the crystallization condition with no biological relevance (Figure 1A).

Molecular recognition by WDR domains occurs via the top, bottom, sides, and central cavity. Specific PPIs are believed to be governed by the sequence insertions outside of the repeats themselves, such as the extended loops that decorate the top and bottom faces. A Dali search was used to determine if such insertions exist in the WDR domain of RFWD3 in comparison to closely related structural homologs. Included in the top hits were WDR5 (WD repeat‐containing protein 5; PDB: 6DAS, LGA score: 69.6) and RFWD2 (PDB: 5HQG, LGA score: 61.3). Alignment of the structures revealed that RFWD3 does indeed contain distinct insertions in the form of disordered loops on the top and bottom faces, specifically encompassing residues 467 to 477, 594 to 606, and 656 to 664 (Figure 1B, panels i‐iii, respectively). Furthermore, elongated strands of repeat five appear to be a feature unique to RFWD3 (Figure 1B panel iv). Interestingly, residues involved in DDR are not part of these insertions, including Trp‐543 and Ile‐639 for binding RPA32, and the QKMDF^624^ consensus motif that mediates the interaction with PCNA (proliferating cell nuclear antigen) during DNA replication.^5^

Many disease‐associated mutations exist within the WDR domain, presenting RFWD3 as a potential anti‐cancer target. The COSMIC (catalogue of somatic mutations in cancer) database was used to identify solvent‐exposed point mutations that may have roles in various cancers (Figure 1C). For example, mutation Cys638Tyr, identified in a whole‐genome screen of colorectal cancers, would abolish the observed Cys638 to Cys696 disulfide (Figure 1A), whereas the FA‐related Ile639Lys mutation will disrupt the hydrophobic packing around Ile639 and likely lead to a destabilized protein. Another interesting feature of this domain is the surface charge distribution about the top and bottom faces (Figure 1D). A large electropositive surface is present on the top face in contrast to a large electronegative surface on the bottom. During DDR, RFWD3 stabilizes p53 by binding Mdm2 (an E3 and negative regulator of p53). We propose that the acidic domain of Mdm2^3^ binds the positively charged top surface of the WDR domain. Indeed, this surface characteristic would also suggest the potential for phosphopeptide recognition, similar to that observed with RFWD2.^9^

How was CASP13 able to predict and model the structure of this important target? Overall, the modeling efforts were successful with 56 predictions (out of 86 total) providing GDT‐TS scores >50. Group A7D was the most accurate structural predictor with a GDT‐TS score of 72.0 and an RMSD of 1.87 å across 2066 atom pairs. Superposition with the crystal structure reveals the overall topology (Figure 1A), including the large disordered loops (Figure 1B), was well reproduced in the model. Importantly, this was achieved despite the notoriously low sequence similarity between homologous WDR domain‐containing proteins.

### 2.2 The H1 domain of human KCTD8 (CASP: T0970, PDB: 6G57). Provided by Daniel M. Pinkas, Joshua C. Bufton, and Alex N. Bullock

KCTD1‐21 form a subgroup of BTB domain‐containing proteins that commonly function as Cullin3‐dependent E3 ligases.^10^ For example, KCTD5 is observed to act as a Cullin3 dependent off‐switch for GPCR signaling through ubiquitin‐mediated degradation of G_βγ_ under certain conditions.^11^ Similarly, KCTD6, KCTD11, and KCTD21 have been observed to ubiquitylate HDAC1 in complex with Cullin3, thereby suppressing Hedgehog activity in Medulloblastoma.^12^ KCTD8, KCTD12, and KCTD16 lack Cullin3 binding and instead act as auxiliary subunits of the GABA_B2_ receptors^13^ that help to modulate signaling outcomes.^14^

KCTD8 consists of an N‐terminal region of 41 amino acids that is predicted to be unstructured, followed by a BTB domain, which mediates interaction with the GABA_B2_ receptor^15^and also axially homo‐ and hetero‐associates with the BTB domains of KCTD12 and KCTD16.^16^ This region is followed by a low complexity region of 54 amino acids and a poorly characterized domain that is conserved between KCTD8, KCTD12, and KCTD16, termed the “H1” domain. C‐terminal to this domain is another region that is homologous between KCTD8 and KCTD16 but missing in KCTD12, dubbed “H2.” The H2 region is predicted to be largely unstructured,^17^ but contains a significant HHpred^18^ signature for a small alpha helical domain with predicted structural homology to a Yeast Mediator of RNA polymerase II (4H62_V) at the C‐terminus of the H2 region.

**Figure 2:**
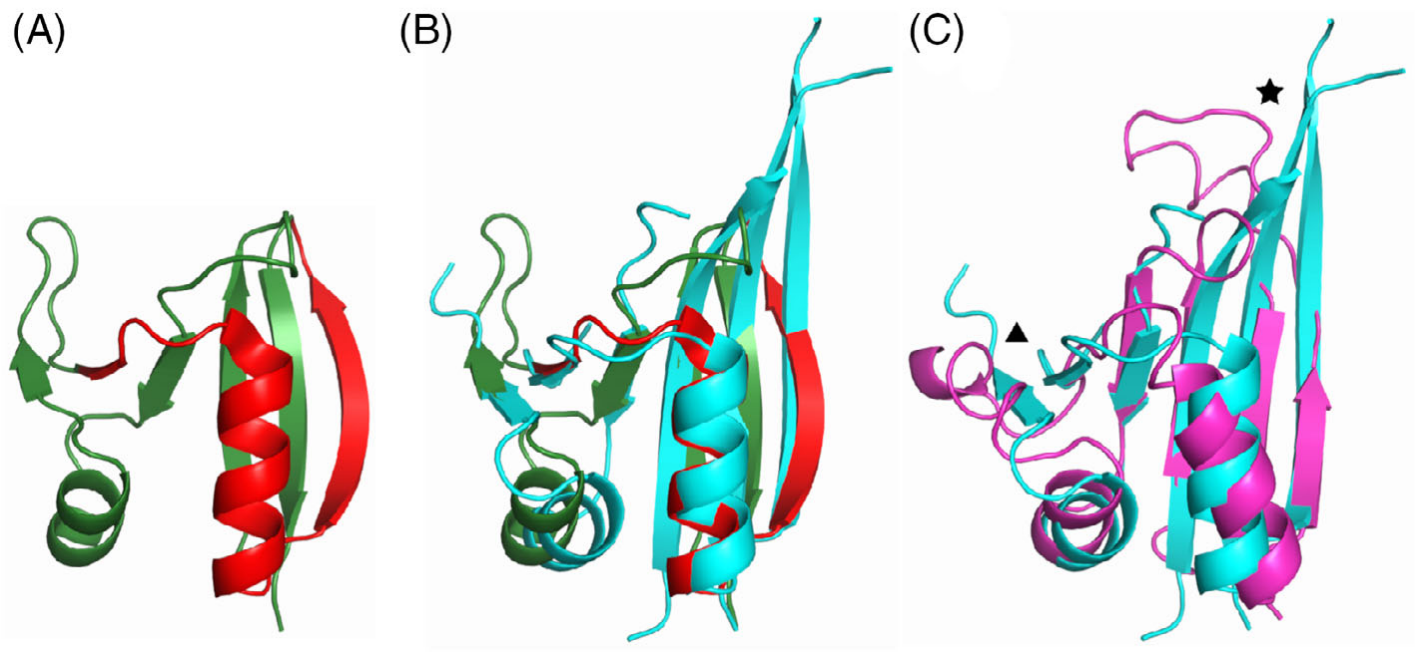
A, Crystal structure of the top HHpred hit (Rat GFRP, PDB: 1JG5_B) for the H1 domain of human KCTD8. Rat GFRP is shown in green. The region matching the H1 domain of KCTD8 is highlighted in red. B, Superposition of Rat GFRP crystal structure (depicted as in Figure 2A) and the crystal structure of human KCTD8 H1 domain (PDB: 6G57, cyan). C, Structure superposition of the crystal structure of human KCTD8 H1 domain (depicted as in Figure 2B) and the top scoring model from CASP13 (T0970TS112_1‐D1, magenta). Two key differences between model and reference are highlighted: a star highlights the region of the C‐terminal beta hairpin structure and a triangle highlights the short beta strand pair

The KCTD8 H1 domain was solved at the SGC and refined to a resolution of 2.75 å (Table 1, Target: T0970; PDB: 6G57, UniProt: Q6ZWB6, construct residues 201‐322). Despite sharing very low sequence identity with any solved crystal structures, a top HHpred hit is detected with a probability score of 77.8% to rat GTP Cyclohydrolase I Feedback Regulatory Protein (GFRP, PDB: 1JG5_B, Figure 2A). The HHpred match only covers 22% of the protein sequence (27/122 residues) and does not correspond to a contiguously interacting folded segment (Figure 2A). Despite the low conservation, the HHpred hit shows a good overall fit to the KCTD8 H1 domain with an LGA score of 58.7 (Figure 2B).^19^

The top scoring model (T0970TS112_1‐D1, GDT‐TS = 67.94) has a similar LGA score of 56.1 (Figure 2C), and faithfully recapitulates some important aspects of the true overall fold, although missing some key features such as an extended C‐terminal β‐hairpin (star) and a short β‐strand pair (triangle) that is conserved between GFRP and KCTD8. However, the extended C‐terminal beta hairpin was correctly predicted in the sixth best overall scoring model (T0970TS043_1‐D1, GDT‐TS = 63.23), and the short beta strand pair correctly predicted in the second best scoring model (T0970TS149_1‐D1, GDT‐TS = 66.18).

Overall, several interesting points arise from analysis of the predicted structures. First, the fact that the GFRP template itself has a slightly higher LGA score than the best solutions raises the question of whether the excellent fit of this template could have been predicted and hence incorporated to generate improved constraints on solutions. Second, although standard high‐throughput multi‐construct design techniques were used to generate the construct that produced the crystal structure of KCTD8, the essentially correct prediction of the H1 domain in this case suggests that current structure prediction techniques could potentially assist in the process of designing expression constructs.

### 2.3 Structure of the human MAJIN‐TERB2 heterotetrameric complex (CASP: H0980, PDB: 6GNX). Provided by Manickam Gurusaran and Owen R. Davies

Meiosis is a two‐stage specialized cell cycle that produces haploid germ cells by reducing the chromosome number by half. It is thus essential for reproduction, genetic diversity, and evolution, with errors in meiosis leading to human infertility, miscarriage and germ cell cancers.^20^ At the center of this process is the establishment of homologous chromosomes pairs through their physical tethering by the synaptonemal complex, and the resultant formation of genetic crossovers.^21^, ^22^ To achieve this requires an ornate choreography in which meiotic chromosomes are rapidly moved around the nucleus to enable the identification of establishment of homologous pairs through meiotic recombination.^23^ These meiotic prophase movements are driven by microtubule forces that are transmitted across the nuclear envelope via the LINC complex, and directed to the telomeric ends of meiotic chromosomes.^24^ In mammals, the meiotic telomere complex (formed by MAJIN, TERB1, and TERB2) physically tethers meiotic chromosome telomere ends both to the inner nuclear membrane and the LINC complex,^25^ thereby permitting microtubule‐driven chromosome end movements within the plane of the nuclear envelope. The molecular architecture of the meiotic telomere complex is defined by a core MAJIN‐TERB2 complex that connects its key functionalities. MAJIN mediates inner nuclear membrane attachment through a transmembrane helix, while TERB2 binds to TERB1, which interacts with shelterin component TRF1 to recruit telomeric DNA, and is also thought to bind to the LINC complex.^25^, ^26^, ^27^ Previous genetic studies in mice demonstrated that individual disruption of MAJIN, TERB1, or TERB2 leads to impaired telomere attachment, failure of chromosome movements and infertility.^25^, ^26^, ^27^, ^28^ We thus initiated structural studies to understand the molecular basis of this essential process of mammalian meiosis.

**Figure 3:**
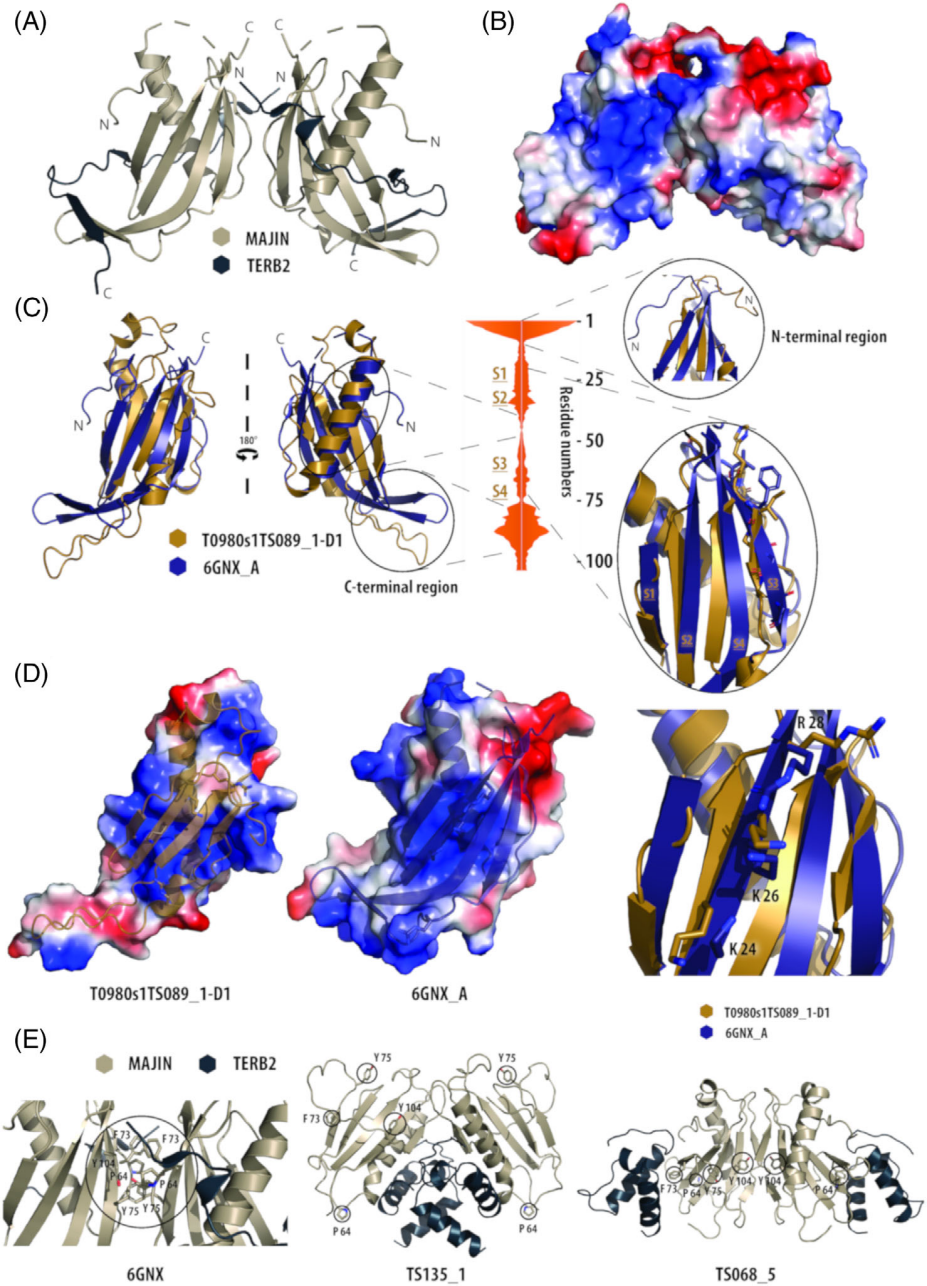
A, Cartoon representation of the crystal structure of MAJIN‐TERB2 illustrating a 2:2 hetrotetrameric complex. B, Surface electrostatic potential (blue: electropositive; red: electronegative) of the MAJIN‐TERB2 displaying an extensive basic surface, which mediates direct interaction with DNA. C, Superposition and residue‐wise RMSD plot of the MAJIN protomer (PDB code: 6GNX, chain A) and T0980s1TS089_1‐D1. D, Surface electrostatic potential (blue: electropositive; red: electronegative) of T0980s1TS089_1‐D1, MAJIN protomer (PDB code: 6GNX, chain A) illustrating the basic patches. Right‐hand panel compares the basic patch on T0980s1TS089_1‐D1 and the target. E, Compares the MAJIN dimer interface with the two best models. Aromatic and proline residues that are essential for dimer stabilization are highlighted

The crystal structure of the MAJIN‐TERB2 core complex (Table 1, Target: H0980; PDB ID: 6GNX) revealed a 2:2 heterotetramer in which two TERB2 chains wrap around a globular MAJIN dimer (Figure 3A).^29^ Each MAJIN protomer adopts a β‐grasp fold, in which a β‐sheet grasps around a core α‐helix (Figure 3A). The structural architecture of the β‐grasp fold consists of a five‐stranded β(2)‐α‐β(3) assemblage with a two‐stranded β‐sheet insertion, which is seemingly unique to MAJIN‐TERB2. The MAJIN dimerization interface is stabilized through aromatic and proline interactions (Figure 3E, left), with additional stabilization from TERB2. The structure displays an extensive basic patch on the surface of each MAJIN protein (Figure 3B), which binds to DNA and thereby provides a novel means for meiotic telomere recruitment. Further, the relative orientation of N‐ and C‐termini of both protein components allowed us to model the architecture of the wider MAJIN‐TERB2‐TERB1 complex.^29^

The category of prediction experiments of a MAJIN monomer included 92 models, of which many successfully predicted a β‐grasp fold (eg, T0980s1TS089_1‐D1, GDT‐TS = 54.81), while others were highly divergent (eg, T0980s1TS458_1‐D1, GDT‐TS = 19.71). Model T0980s1TS089_1‐D1 most closely resembles the MAJIN protomer of the crystal structure, with a TM‐score of 0.54 (GDT‐TS = 54.81), and so is discussed presently. Superposition of model T0980s1TS089_1‐D1 onto the MAJIN protomer structure (PDB code: 6GNX, chain A) demonstrates that the topology of the fold was predicted with an impressive level of accuracy (Figure 3C). The core α‐helix was predicted with local C_α_ RMSD of ~1 å and interacts with the grasping β‐sheet through largely native contacts; the β‐strands are similarly predicted correctly, although the angulation between α‐helix and β‐sheet deviates slightly from then native structure (Figure 3C). The main divergent regions of the model are the MAJIN N‐termini and two‐stranded β‐sheet insertion. MAJIN N‐termini lack secondary structure and form surface hydrophobic contacts with the remainder of the structure (Figure 3C). While the β‐sheet insertion correctly links between strands of the grasping β‐sheet, its conformation and orientation differ from the crystal structure, although it is possible that the conformation of this region is stabilized by crystal lattice (Figure 3C). Importantly, the model shows similar electrostatic properties along the MAJIN DNA binding surface (Figure 3D).

In the category of oligomeric modeling, there were 73 predictions, of which none correctly modeled the MAJIN‐TERB2 2:2 complex. A number of models accurately predicted the core of the MAJIN β‐grasp fold but failed to predict the MAJIN dimer interface, which involves amino acids Pro64, Phe73, Tyr75 (Figure 3E, left). In some cases, the overall MAJIN dimers show superficial similarity with the crystal structure, but with incorrect β‐grasp topologies placing residues Pro64, Phe73, Tyr75 far from the interface (eg, TS068_5; Figure 3E, right). In other cases, the interface shows no resemblance to the crystal structure (eg, TS135_1; Figure 3E, mid). Modeling of TERB2 was consistently aberrant as it was typically predicted to adopt a small globular fold that binds to MAJIN, in stark contrast to its extended conformation wrapping around a MAJIN protomer, through a series of surface hydrophobic and β‐sheet interactions in the crystal structures. Components of a constitutive complex, such as MAJIN‐TERB2, likely undergo a coordinated folding process in vivo that results in their co‐dependence for stability. This likely highlights an important challenge in modeling, that in such cases it is inappropriate to predict oligomers through modeling of prefolded protomers, and instead requires co‐folding of multiple chains in silico.

### 2.4 Crystal structure of LP1413, an unusual single‐stranded DNA binding protein (CASP: T0958, PDB: 6BTC). Provided by Ignacio Mir‐Sanchis and Phoebe A. Rice

We named this protein LP1413 as it is a little protein (96 amino acids) annotated as containing DUF (domain of unknown function) 1413.^30^ We were interested in its structure and function as part of our ongoing project to understand the SCC family of mobile genomic islands, many of which carry methicillin resistance. Insertion of these elements into the *Staphylococcus aureus* chromosome creates MRSA (methicillin‐resistant *S. aureus*) strains. We have defined the set of core conserved genes carried by these highly mosaic mobile elements, and are working to determine their functions.^31^

LP1413 is encoded in the same operon as a helicase, Cch, that has sequence homology to replication initiator proteins from a different family of mobile elements, the SaPIs, and that has structural homology to MCM helicases.^31^ We detected no enzymatic activities in purified LP1413 but found that it binds single‐stranded DNA with high affinity. The protein was monomeric in solution^30^ (Table 1, Target: T0958).

**Figure 4:**
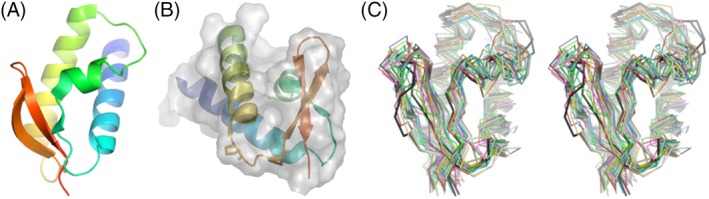
Crystal structure and models for LP1413. A, Ribbon diagram of the crystal structure, shaded from blue (N‐terminal) to red (C‐terminal). B, The crystal structure from a slightly different viewpoint, with a transparent molecular surface showing the hydrophobic pocket (roughly center) and the conserved Proline residues of the helix 3––strand 2 turn shown as sticks. C, Superposition of the top 40 predicted models (wall‐eyed stereo), from the same viewpoint as in panel A. The two chains from the asymmetric unit of the crystal structure are shown in black

We thought that LP1413 might be an interesting CASP target because our crystal structure shows it to be a winged helix‐turn‐helix domain (Figure 4A), but it was not annotated as such in sequence databases. Also, the structure has two unusual features: a β‐bulge in strand 2, and an unusually long turn between helix 3 and strand 2 hosting conserved prolines which helps create a small hydrophobic pocket (Figure 4B). In the crystal, M1 of an adjacent monomer is inserted into this pocket. However, we found that an M1G change caused almost no change in affinity or cooperativity in binding single‐stranded DNA, so the natural ligand for this pocket remains unknown.

Among the highest ranked models according to GDT‐TS, all predicted the correct overall fold except one (T0958TS124_1‐D1, ranked third with GDT‐TS = 74.03) where the order of beta strands 2 and 3 was reversed. Overall, the models diverged most in the placement of the shortest helix, helix 2, and the turn between helices 2 and 3. Except for the poorly ordered N‐ and C‐termini, which were not included in the prediction contest, the backbone atoms of that turn had the highest backbone B‐factors in the model, and was the one region where the two copies in the asymmetric unit diverged slightly. This suggests, not surprisingly, that flexibility correlates qualitatively with difficulty in prediction.

Only 6 of the top 40 models correctly predicted the β‐bulge at Val68. In terms of overall GDT‐TS scores, they were near both the top and the bottom: rank 1, 2, 5, 28, 30, and 39. The top two scoring models also contained the best predictions for the conformation of the helix 3––strand 2 turn. These two models, both from the Laufer group, also at least partially predicted the hydrophobic pocket, although this feature was hard to score objectively. Overall, 39 of the top 40 models predicted the correct fold, and the top two were remarkably correct in detail. Ironically, had we known that this protein adopts a winged helix‐turn‐helix fold, we would have guessed double‐ rather than single‐stranded DNA binding as its function.

### 2.5 Contact‐dependent growth inhibition toxin‐immunity protein complexes from *E. coli* 3006 (CASP: H0957, PDB: 6CP8) and *K. pneumoniae* 342 (H0968, PDB: 6CP9). Provided by Karolina Michalska, Christopher S. Hayes, Celia W. Goulding, Andrzej Joachimiak

Bacteria use several mechanisms to communicate, cooperate, and compete with neighboring microbes in the environment. In dense communities, bacteria use a number of secretion systems to deliver protein toxins directly into their competitors.^32^ This phenomenon was first discovered in *E. coli* isolate EC93 and was termed “contact‐dependent growth inhibition” or CDI.^33^ CDI is mediated by the CdiB and CdiA two‐partner secretion proteins, which form a complex on the cell surface.^33^ CdiA is a filamentous protein that extends several hundred angstroms to interact with receptors on the surface of susceptible target bacteria. Upon binding its receptor, CdiA undergoes a series of conformational changes that ultimately deliver its C‐terminal toxin domain (CdiA‐CT) into the target cell.^34^ To protect themselves from self‐intoxication, CDI^+^ cells also produce a CdiI immunity protein that binds the CdiA‐CT to neutralize toxin activity. Though characterized most extensively in *E. coli*, CDI systems are broadly distributed throughout Gram‐negative bacteria including pathogens^35^ and have been implicated in cooperative behaviors, such as biofilm formation, persistence, and virulence.^36^, ^37^, ^38^ CdiA effectors carry extraordinarily diverse CdiA‐CT regions, indicating that the systems deploy many distinct toxins. Similarly, CdiI sequences are also highly variable, and each immunity protein only provides protection against its cognate toxin. Thus, CDI toxin‐immunity protein polymorphism underlies an important mechanism of self/nonself discrimination in bacteria.

**Figure 5:**
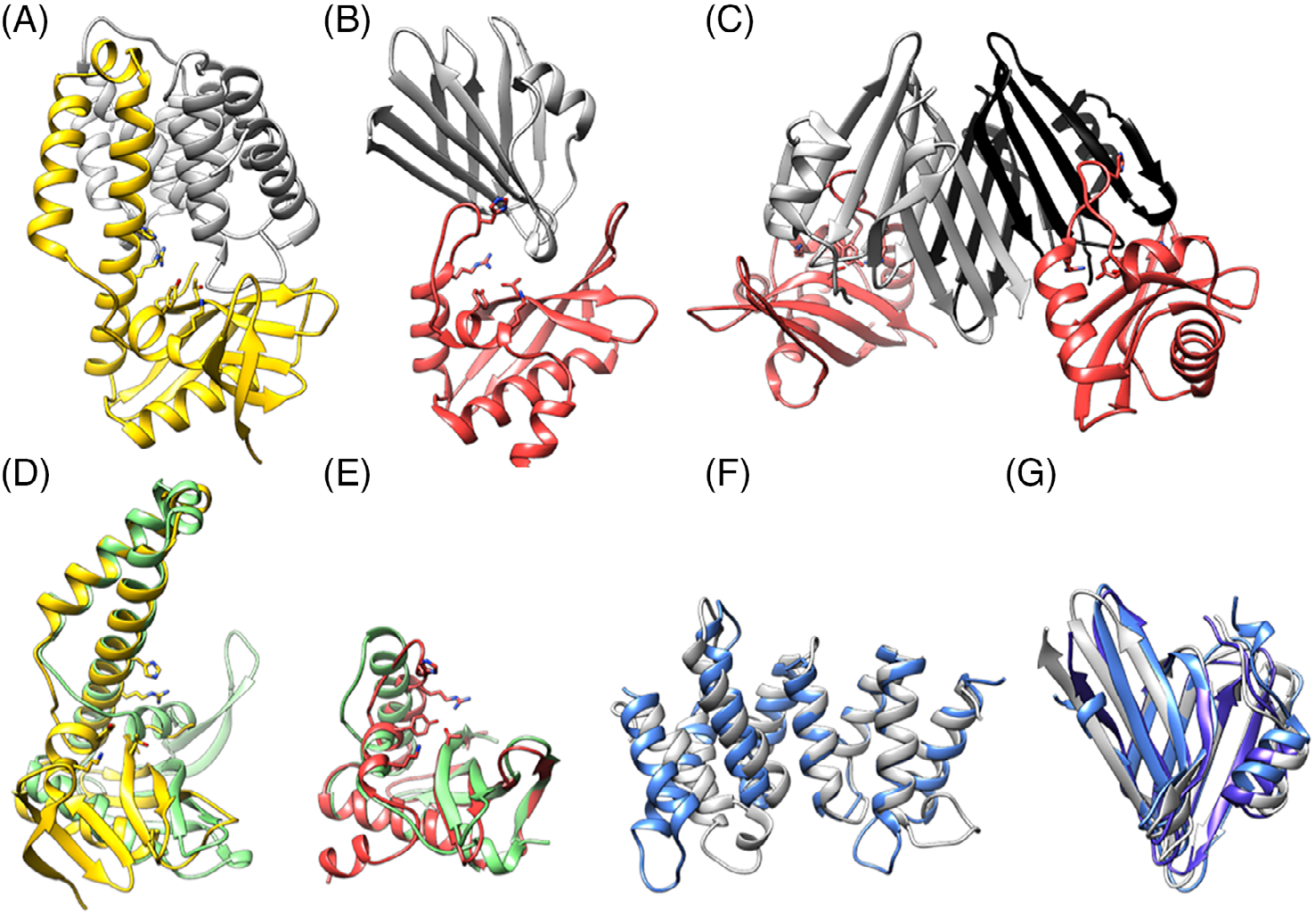
A, Experimental structure of CdiA‐CT•CdiI^Ec3006^. The CdiA‐CT^Ec3006^ toxin domain is shown in yellow with functionally important residues shown in stick representation. The CdiI^Ec3006^ immunity protein is shown in gray. B, Experimental structure of the CdiA‐CT•CdiI^Kp342^ (heterodimer) with CdiA‐CT^Kp342^ shown in red and CdiI^Kp342^ in gray/black. C, Experimental structure of the CdiA‐CT•CdiI^Kp342^ (hetero‐tetramer) with CdiA‐CT^Kp342^ shown in red and CdiI^Kp342^ in gray/black. D, Superposition of CdiA‐CT^Ec3006^ (yellow) with T0957s1TS043_4 (green). E, Superposition of CdiA‐CT^Kp342^ (red) with T0968s1TS043_4‐D1 (green). F, Superposition of CdiI^Ec3006^ (gray) with T0957s2TS043_5‐D1 (blue). G, Superposition of CdiI^Kp342^ (gray) with T0968s2TS043_1‐D1 (blue) and T0968s2TS214_2‐D1 (purple)

CDI toxin‐immunity protein complexes are excellent targets for methods development in the CASP competition, because the activities of most toxins are unknown and their interactions with cognate immunity proteins are not easily predicted. Over the past several years, we have taken complementary biochemical and structural approaches to identify CDI toxin activities and explore the diversity of their interactions with immunity proteins.^39^, ^40^, ^41^, ^42^, ^43^ We recently solved the crystal structures of CdiA‐CT•CdiI complexes from *E. coli* 3006 (EC3006) and *K. pneumoniae* 342 (Kp342) at 2.20 and 2.55 å resolution. The CdiA‐CT^EC3006^ toxin consists of a globular α/β core and an extended α‐helical subdomain (Figure 5A). The core is composed of two 3‐stranded antiparallel β‐sheets that interact in parallel and wrap around helix α5, which forms the spine of the subdomain (Figure 5A). The extension subdomain comprises three α‐helices that mediate many of the direct contacts with CdiI^EC3006^ (Figure 5A). CdiA‐CT^Kp342^ shares a number of structural elements with the CdiA‐CT^EC3006^ core; though the CdiA‐CT^Kp342^ domain lacks the N‐terminal β‐sheet, and its C‐terminal β‐sheet consists of four strands (Figure 5B). Further, the α‐helical extension subdomain is abbreviated to one helix and a loop in CdiA‐CT^Kp342^. Though the toxins share significant structural homology, the immunity proteins have completely unrelated structures. CdiI^EC3006^ is an α‐helical monomer that adopts an α‐solenoid fold (Figure 5A), whereas CdiI^Kp342^ forms a dimeric β‐sandwich (Figure 5C).

The structures reveal that both toxins are members of the barnase/EndoU/colicin E5‐D/RelE (BECR) superfamily of RNases,^44^ though they exhibit no detectable sequence similarity to BECR enzymes and are not annotated as such. DALI identifies the C‐terminal nuclease domain of colicin D as the closest structural homolog of CdiA‐CT^Kp342^. CdiA‐CT^Kp342^ residues Lys157, Tyr160, and Thr255 superimpose onto the active site residues of colicin D. Furthermore, CdiA‐CT^EC3006^ contains a similar triad of Lys204, Tyr208, and Thr330 arranged in the same configuration. The toxins also share conserved Arg and His residues that could play roles in binding substrate and catalysis. Previous activity studies have shown that CdiA‐CT^EC3006^ specifically cleaves tRNA^Ile^ molecules,^45^ indicating that the toxin is indeed a BECR family RNase. Notably, the CdiI proteins both bind over the predicted active sites of their cognate toxins (Figure 5A,B), indicating that the immunity proteins neutralize toxicity by blocking access to RNA substrates.

For the CASP13 competition, the components of each CdiA‐CT•CdiI complex were first modeled as monomers, and the top 10 predicted models according to GDT‐TS were evaluated (Table 1, Target: H0957). For CdiA‐CT^EC3006^, the best five predictions have GDT‐TS scores ranging from 49.07 (T0957s1TS043_4) to 45.22 (T0957s1TS043_1). These models predict the existence of the C‐terminal β‐sheet, the spine α‐helix, and the α‐helical extension subdomain (Figure 5D). The N‐terminal β‐sheet and following α‐helix of CdiA‐CT^EC3006^ are positioned incorrectly, displacing the C‐terminal β‐sheet from its position in the experimental structure. This results in the high global deviation for the top model T0957s1TS043 with a GDT‐TS of 49.07 (Cα RMSD = 13.80 å); local agreement of 1.77 å is attained for only 47% of residues within a 5 å distance cutoff. The sixth model (T0957s1TS117_4, GDT‐TS = 39.97 and RMSD = 10.69 å) includes the α‐helical extension, but fails to capture the core α/β subdomain. Neither of the β‐sheets is properly generated, and the two α‐helices are not positioned correctly. The remaining four models are essentially identical (GDT‐TS = 38.73) and bear little similarity to the experimental structure. Finally, none of the models arrange the putative active site residues properly.

The top 10 CdiA‐CT^Kp342^ models are very similar to one another and more accurately reflect the experimental structure (Table 1, Target: H0968; Figure 5E), with GDT‐TS scores ranging from 77.54 (T0968s1TS043_4‐D1, global RMSD_Cα_ 5.04 å) to 69.92 (T0968s1TS043_2‐D1, global RMSD 4.53 å). Some deviations are observed at the N‐terminus and the protruding α‐helix (Figure 5E). Locally, the top model aligns within 1.71 å over 85% of residues. These results suggest that the structure of the isolated CdiA‐CT^Kp342^ toxin domain is very similar to that observed in complex with CdiI^Kp342^.

The CdiI^EC3006^ models mirror the crystallographic structure quite well, with GDT‐TS scores of 65.32 to 60.65. All of the models feature consecutive α‐hairpins and differ only in the placement of the C‐terminal α‐helix and the α1‐α2 loop, for which none of the models show good alignment with the target (Figure 5F). We note that the positions of these latter elements may be constrained through interactions with the CdiA‐CT^EC3006^ toxin domain. The top model (T0957s2TS043_5‐D1, GDT‐TS = 65.32) yields an overall C_α_ RMSD of 4.15 å and local of 2.11 å over 68% of residues.

More accurate predictions were obtained for CdiI^Kp342^, with the 10 best models scoring GDT‐TS of 78.70 to 71.30. As with CdiI^EC3006^, the major deviations were localized to the N‐ and C‐terminal regions, which the top model (T0968s2TS043_1‐D1; GDT‐TS = 78.70, 2.33 å global C_α_ RMSD and 1.77 å over 96% residues) misrepresents as helical turns (Figure 5G). It is the third model (T0968s2TS214_2‐D1; GDT‐TS = 77.39, 2.05 å global C_α_ RMSD and 1.81 å over 92% residues) that correctly predicts a random coil and a β‐strand at the termini. Again, the latter section is in close proximity to the toxin domain in the complex structure.

As we found during CASP12, the prediction of protein‐protein binding interfaces remains a significant challenge. Even with decent monomeric models and supporting information from SAXS and cross‐linking data for CdiA‐CT•CdiI^Kp342^, no meaningful theoretical complex was generated. This is perhaps because the best monomer predictions were not used to predict the complex structure. For example, CdiA‐CT^Kp342^ from the highest scoring CdiA‐CT•CdiI^Kp342^ model (H0968TS208_4) ranked 114th in the individual subunit calculations, with a GDT‐TS = 55.09. Another model of a complex, H0968TS163_2, features reasonably good CdiA‐CT (T0968s1TS163_1‐D1, GDT‐TS = 69.28), and wrongly predicted CdiI (T0968s2TS163_2‐D1, GDT‐TS 42.39), even though the same group had a much better model in their repertoire (T0968s2TS163_4‐D1, GDT‐TS = 61.74). Generally, oligomeric predictions vary enormously and the algorithms struggle to identify the proper interacting surfaces. Analysis of any structural details and their functional implications appears to be beyond the reach of current computational approaches, at least for these particular targets.

### 2.6 A putative ACAD from *B. bacteriovorus* (CASP: T0961, PDB: 6SD8 (Apo form), PDB: 6SDA (Holo form)). Provided by Christopher J. Harding and Andrew L. Lovering

Acyl‐CoA dehydrogenases (ACADs) are a large and important class of metabolic enzymes with diverse functions. ACADs typically catalyze the α,β‐dehydrogenation of various CoA‐fused substrates, linked to the β‐oxidation cycle and amino acid metabolism.^46^ In contrast to traditional roles, recent studies have highlighted a number of ACAD homologs that have important physiological functions beyond β‐oxidation, such as responses to environmental stresses, DNA repair, and adaptation to heat.^47^, ^48^, ^49^, ^50^

Here we investigated a putative ACAD, Bd2924, from the obligate predatory bacterium, *B. bacteriovorus*. Our initial interest arose in Bd2924 when it was first identified as a potential novel cyclic di‐GMP (cdG) interacting protein.^51^ CdG is an almost universally important bacterial secondary messenger molecule influencing growth and a range of behaviors such as motility, virulence, biofilm formation, cell cycle progression and (in *Bdellovibrio*) control of predation.^52^

**Figure 6:**
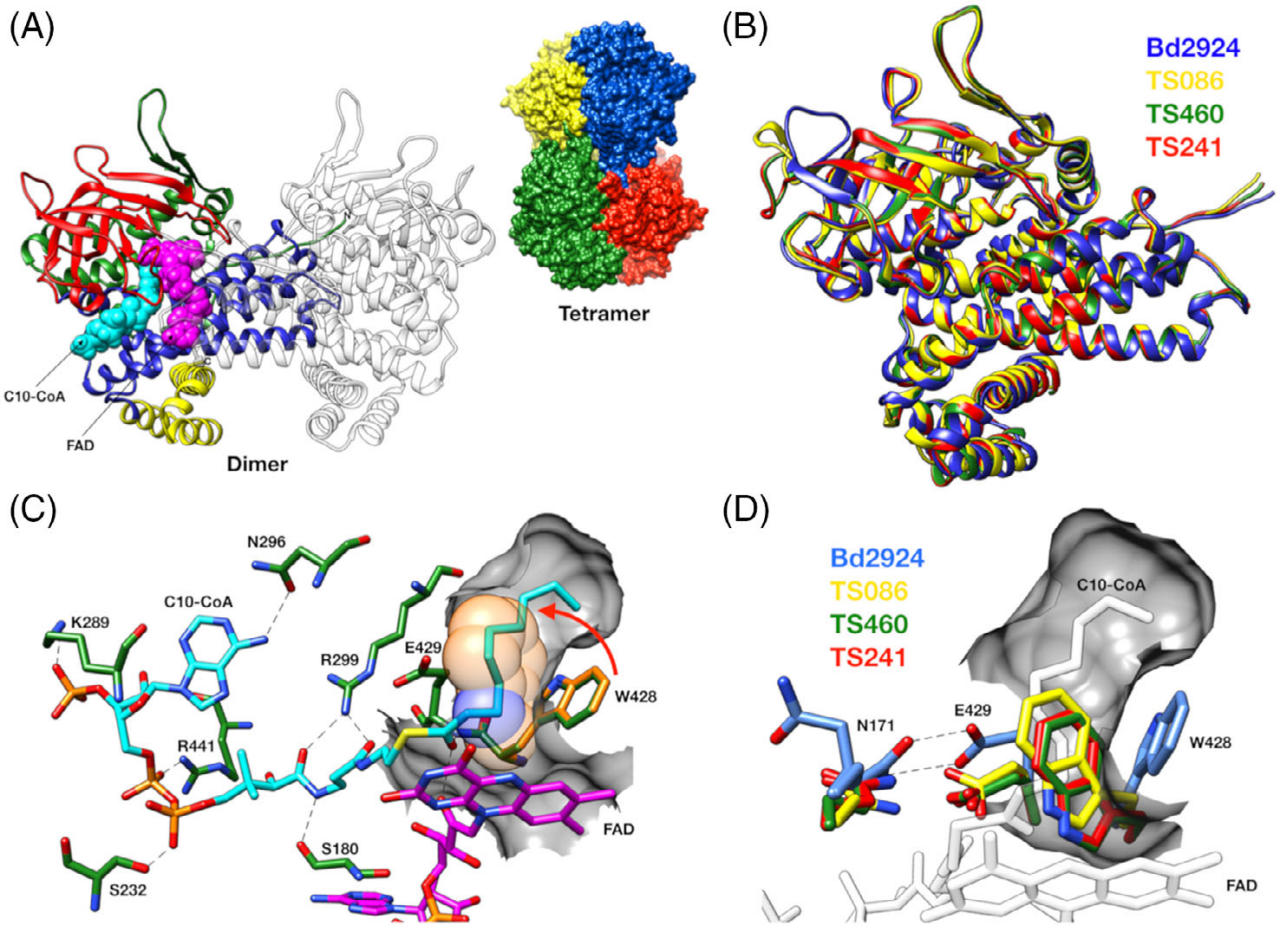
A, Crystal structure of Bd2924 showing 4 distinct domains: a N‐terminal domain α‐helical domain (green, residues 3‐168); a central β‐sheet domain (red, 169‐283); a C‐terminal α‐helical domain (blue, 286‐449) and a C‐terminal extension domain (yellow, 450‐505). FAD cofactor (magenta) and C10‐CoA ligand (cyan) are shown in spacefill. B, Superposition of Bd2924 crystal structure (blue) and the three best models: T0961TS086_1 (yellow), T0961TS460_1 (green), T0961TS241_1 (red). C, A close‐up view of the C10‐CoA ligand binding site (cyan). The CoA moiety makes 8 hydrogen bonds (black dashed lines) and other non‐bonding interactions, while the fatty acyl chain inserts into a narrow tunnel (gray surface). Access to the binding tunnel appears to be gated by the conformation of W428 (orange), which adopts two conformations; one that allows access (C10‐CoA present) and one that blocks access (C10‐CoA absent). D, Comparison of the active site between Bd2924 crystal structure and the three best models (same coloring scheme as in Figure 6B)

We determined the structure of Bd2924 (residues 3 to 505) in complex with the cofactor FAD, with and without a C10 length acyl‐CoA thioester ligand (C10‐CoA) bound, to 1.51 and 1.87 å respectively (Table 1, Target: T0961). The tertiary structure of Bd2924 forms a biologically relevant homo‐tetramer, composed of a dimer of dimers, which was later confirmed to be the predominant species in solution by size exclusion chromatography experiments. Bd2924 shares the common ACAD fold,^53^ and is most closely related to the divergent ACADs, AidB, and ACDH‐11.^49^, ^50^, ^51^, ^52^, ^53^ Bd2924 can be divided into four distinct domains: an N‐terminal α‐helical domain that is interrupted by a single β‐sheet projection (residues 3‐168), a central β‐sheet domain formed by two orthogonal four‐stranded antiparallel β‐sheets (residues 169‐285), a central α‐helical domain (residues 286‐449) and a short C‐terminal α‐helical domain (residues 450‐505) (Figure 6A). A single FAD molecule binds per monomer in a crevice located at the dimer interface. The Bd2924:C10‐CoA complex provided structural insights into the interactions Bd2924 makes with its substrate. The C10‐CoA ligand bound into a long narrow tunnel that runs deep into the protein beneath the bound FAD molecule, similarly to that described for other ACAD structures.^46^, ^47^, ^48^, ^49^, ^50^, ^51^, ^52^, ^53^ Interestingly, only one molecule of C10‐CoA per dimer could be confidently placed into the electron density maps, which corresponded to the conformation of Trp428 beneath the *re*‐face of the FAD molecule. In our structures, Trp428 appears to gate accessibility for the acyl moiety (Figure 6C). Analysis of Bd2924 did not reveal any conventional cdG binding sites in the structure, nor was a cdG complex obtainable. Further biophysical binding experiments were conducted, which suggested weak non‐specific binding between cdG and Bd2924 (data not shown).

The tetrameric assembly of divergent ACADs appears to block the proposed docking site of electron transferring flavoprotein (ETF).^54^, ^55^ Interestingly, we were unable to detect any dehydrogenation activity; likewise no significant dehydrogenase activity has been reported for other divergent ACADs.^49^, ^51^, ^52^, ^53^, ^54^, ^55^, ^56^ Moreover, the structure of Bd2924 reveals that the chemistry of the conserved active site residue Glu429 may be altered by participating in a hydrogen bond with Asn171. Typically a hydrophobic residue such as phenylalanine is found at the position of Asn171 in “conventional” catalytically active ACADs.

Bd2924 was included in CASP13 as target T0961 and also selected for CAPRI experiments. In general, models predicted the overall fold of Bd2924 to a high standard and included most of the main features identified from our crystal structure (Figure 6B). This may not be entirely surprising, considering the highly conserved fold within the ACAD superfamily and the presence of highly similar homologs (AidB and ACDH‐11) in the PDB that could act as templates. Notably, the models were able to correctly predict the structural features of divergent ACADs and the tetrameric assembly. The models also predicted the large groove in the C‐terminal domain, which was a noteworthy feature of Bd2924s structure. A loop region (residues 191‐202) contained the least similarity to our experimental structure and had the most variability in the top models. This is likely due to the loop being a relative insertion in the primary sequence of Bd2924 in comparison to templates AidB and ACDH‐11. Specifically, models deviated around the ligand binding and active site regions (Figure 6D). For instance, the modeled conformation of Trp428 obstructs the depth of the substrate‐binding tunnel, which would lead to incorrect predictions about the length of fatty acyl chains that can be accommodated. Furthermore, the strictly conserved catalytic glutamate residue, Glu429, is also modeled in various conformations. Our crystal structure highlighted a potentially important hydrogen bond between Glu429 and Asn171, which may explain the lack of observable catalytic activity. However, none of the top models successfully model the same hydrogen‐bonding interaction captured in the crystal structure. A major drawback to the models is the lack of precision regarding Bd2924 interaction with its cofactor FAD, which is an integral part of the structure of ACADs.^53^

### 2.7 The receptor‐binding tip (gp37_3_‐gp38) from the *Salmonella* phage S16 long tail fiber (H0953, PDB ID: 6F45). Provided by Matthew Dunne and Petr G. Leiman

**Figure 7:**
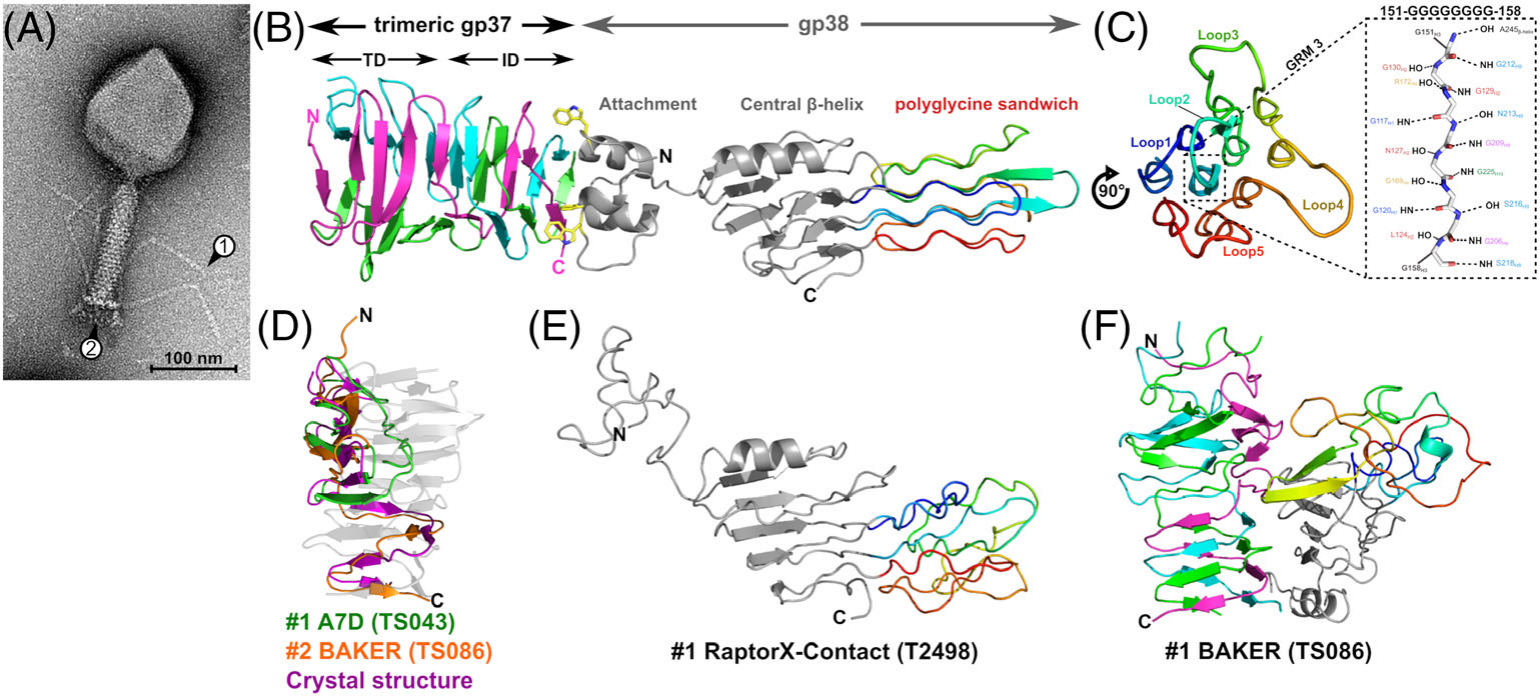
The adhesin tip of the *Salmonella* phage S16 long tail fiber. A, Transmission electron micrograph of phage S16 with arrows (1) pointing to the approximate location of gp38 at the tip of the LTF and (2) pointing to the baseplate. B, Cartoon representation of the LTF distal tip complex of homotrimeric gp37 β‐helix (cyan, magenta, pink) attached to a single gp38 adhesin (gray) with the structurally unique “polyglycine sandwich” domain rainbow colored (blue to red). Gp38 connects to gp37 through hydrophobic interactions, involving three highly conserved tryptophan residues on the apex of each α‐helix of the gp38 attachment domain (yellow sticks) that occupy three symmetry‐equivalent hydrophobic pockets on the gp37 base. C, Head‐on view of the polyglycine sandwich domain formed by the 10 glycine‐rich motifs (GRMs) of gp38 folded into a three‐layered lattice of PG_II_ helices. Labeled are the five distal loops formed by HVSs that form the (yet unknown) receptor‐binding site(s) of gp38. Highlighted by the dashed box is a cartoon representation of the saturating hydrogen‐bonding network of the central GRM 3 with main‐chain carbonyl (CO) and amide (NH) moieties of neighboring helices. D, Superposition of the two best model predictions of monomeric gp37 (T0953s1‐D1) by groups A7D (GST_TS: 54.48) and BAKER (GST_TS: 48.88). E, Cartoon view of the best prediction for monomeric gp38 with residues expected to form the distal polyglycine sandwich rainbow colored (GST_TS: 40.12). F) The overall best prediction for the whole gp37‐gp38 multimer (QS_global_: 0.368, BAKER group). Panel A is reproduced with permission from Dunne M, Denyes JM, Arndt H, Loessner MJ, Leiman PG, Klumpp J. Salmonella phage S16 tail fiber adhesin features a rare polyglycine rich domain for host recognition. *Structure*. 2018;26(12):1573‐1582 e1574

Bacteriophages (phages), viruses that infect bacteria, have served as indispensable tools for many generations of scientists, in particular for discovering the nature and structure of genetic material^57^, ^58^ and more recently for the discovery of the CRISPR system.^59^, ^60^ Phages and their component parts are also used as diagnostic tools and remediative agents in food processing, biotechnology, and medicine (ie, phage therapy).^61^, ^62^, ^63^ The majority of phages consist of an icosahedral “head” (or capsid) containing a dsDNA genome, connected to a specialized delivery organelle called the “tail” (Figure 7A). The tail provides selective recognition and attachment to suitable host cells, generating a conduit between the capsid and the bacterial cytoplasm through which the phage genome is delivered. The initial binding of phage to a bacterial cell is mediated by fibers or stockier “tailspikes” proteins emanating from a baseplate structure at the end of the tail. While internal host defense systems (eg, CRISPR systems) can inhibit phage infectivity, the spectrum of cell surface receptors to which fibers and tail spikes recognize remains the primary determinant of host range for phages. To this end, phage S16 is special as it can infect many *Salmonella* strains, suggesting that either S16 recognizes a wide assortment of cell surface substrates, or S16 targets a highly conserved receptor of *Salmonella*. S16 is a relative of the well‐studied phage T4. Both phages are equipped with baseplate‐attached long tail fibers (LTFs) that mediate receptor‐binding through their distal tips.^64^, ^65^, ^66^, ^67^, ^68^ We recently exploited the *Salmonella‐*specific binding of the S16 LTF as a tool for rapid, ELISA‐like detection of *Salmonella* contaminants in food.^69^

The T4 and S16 LTFs are similar to each except for the structure of their distal tip that interacts with the host cell surface during host recognition. The distal tip of the T4 LTF is formed by the C‐terminal domain of gene product 37 (gp37), whereas the S16 LTF carries an additional protein––gp38 that caps gp37. Gene 38 is present in the T4 genome, but the amino acid sequence of T4 gp38 is very different from that of S16 gp38 and its function is to assist folding and assembly of the T4 LTF. Ironically, the prototypical and better studied T4 phage in which gp38 does not participate in host recognition is a less common representative of T‐even phages most of which appear to carry S16‐like LTFs. Unsurprisingly, the structure and function of gp38 and its homologs (commonly named “adhesins”^70^) have been of great interest ever since they were discovered as the determinants of phage host range.^71^ Thus, by solving the crystal structure of a distal part of gp37 connected to gp38 from phage S16 we aimed to advance our understanding of this important family of adhesins (Figure 7B).^68^

Gp37 is a homotrimeric β‐helix. Five β‐strands (β1 to β5) of each chain form a concave, antiparallel β‐sheet arranged into a three‐sided β‐prism called a “triangular domain.” The following β6 to β9 maintain the β‐helix topology, however, successive strands alternate between the three sides and form an “interdigitated domain.” The N‐terminal helical bundle (α1‐α3) of gp38 attaches to the base of the interdigitated domain through strong hydrophobic interactions. At the apex of α1 to α3 are exposed tryptophan residues that insert every 120° around the fiber axis into similar hydrophobic pockets on the sides of the gp37 β‐helix. These tryptophans and other hydrophobic interface residues are conserved in the adhesins of phages targeting different hosts, suggesting a preference for this mode of attachment in other fibers. A short linker connects the N‐terminal domain to a central five‐ringed β‐helix. Here, all β‐strands and turns align perpendicularly to the fiber axis. Strikingly, the two turns connecting β5 to β6 and β8 to β11 of the β‐helix (18 and 77 residues, respectively) extend along the fiber axis and form the distinct, three‐layered “polyglycine sandwich” domain. Over 50% of residues within this domain are glycines (37 out of 73 residues) distributed between 10 glycine‐rich motifs (GRMs) separated by short hypervariable segments (HVSs). The GRMs all form left‐handed, elongated helices of a rare polyglycine type II (PG_II_) disposition (Figure 7C) with similar properties to polyproline type II (PP_II_) helices, as found in collagen.^72^ The shorter β5 to β6 extension forms the first two helices (1‐2) and the longer β8 to β11 extension generates the last eight helices (3‐10). Connecting the helices are the HVSs that form short β‐turn loops at both ends of the domain. Interestingly, sequence variability within the five distal HVS loops (Figure 7C) directly relates to host range adaptation, and is therefore where we propose receptor binding occurs.

To the best of our knowledge, only four other structures in the PDB contain similar PG_II_ motifs; however, none of them feature long GRMs (eg, helix 3; 151‐GGGGGGGG‐158) or form similar lattices as the polyglycine sandwich. The absence of similar structures within the PDB makes the polyglycine sandwich an especially difficult domain to predict. As expected, the combination of this structurally unique domain and the unusual composition of the (gp37)_3_‐gp38 complex, particularly the trimer‐monomer interface, proved an especially challenging target in CASP (Table 1, Target: H0953). For instance, gp38 alone was modeled poorly and only the top prediction, T0953sTS498_1 by the RaptorX‐contact group, produced a GDT‐TS score > 40. Nevertheless, upon visual inspection the top three predictions all roughly determined the correct gp38 topology and its three distinct domains. As shown for the top model (Figure 7E), the central β‐helix is predicted quite well and includes the capping α‐helix (α4) as well as the important two loop extensions that form the polyglycine sandwich in the crystal structure. Interestingly, in this model the loops generate a similar sandwich‐like structure, which correctly features connecting β‐turn loops at either end; however, the intricate lattice composition of the polyglycine sandwich is missing. As seen with this model, the gp38 central β‐helix as a single target (T0953s2‐D2) was also generally predicted well, with 37 out of 90 predictions generating GDT‐TS scores >40.

The highest ranked model for the whole gp37‐gp38 multimer was TS086_1 by the BAKER group (Figure 7F) with a QS‐score of 0.37. Despite failing to predict gp38 and its attachment to gp37 correctly, this model very accurately determined the composition of the gp37 β‐helix, including the N‐terminal triangular and C‐terminal interdigitated domains. As a single target, gp37 (T0953s1‐D1) was similarly predicted well, with the top two models by groups A7D and BAKER (GDT‐TS of 54.48 and 48.88, respectively) shown in Figure 7D. Visual inspection showed correct prediction of the triangular domain; however, only the BAKER group correctly determined the continuation of the gp37 chain to form the interdigitated domain. Interestingly, the next eight best predictions also correctly modeled the triangular domain, but similar to A7D, incorrectly assumed that the C‐terminal part folds back on itself. Possibly, the trimeric nature of the protein was not taken into account for many of these predictions.

To assist the predictions, SAXS and SANS envelopes as well as protein cross‐linking data of the complex were provided to the competition (S/A/X0953). The molecular envelopes generated by SAXS and SANS reproduced the shape of the gp37‐gp38 crystal structure very well; however, it is unclear whether and how these data were used by the CASP participants as the composition of predicted models did not made biological sense or present folds similar to the crystal structure. In fact, models obtained during the regular prediction round without using these envelopes better represented the crystal structure. Due to a lack of potential cross‐linking reactive residues (Lys, Asp, and Glu) within the gp37‐gp38 interface, protein cross‐linking was unfortunately not of assistance with oligomeric predictions.

### 2.8 Pentafunctional AROM Complex from *Chaetomium thermophilum* (CASP: T0999, PDB: NA). Provided by Harshul Arora Veraszto and Marcus D. Hartmann

The AROM complex is a homodimeric pentafunctional fusion enzyme in the shikimate pathway in fungi and protists.^73^ This pathway is a seven‐step biosynthetic route to chorismate, the central precursor for aromatic amino acids and other aromatic compounds,^74^ and a textbook drug target for being essential in prokaryotes and lower eukaryotes, but absent from metazoa. Most prominently, it is the target of glyphosate, the active ingredient of Monsanto's ubiquitous blockbuster weed‐killer Roundup.^75^ While most organisms have the seven steps of the pathway encoded as individual, monofunctional enzymes, fungi, and protists have the five central steps fused in the pentafunctional AROM polypeptide. These five steps comprise the 3‐dehydroquinate synthase (DHQS),^76^ 3‐dehydroquinate dehydratase (DHQD),^77^ shikimate dehydrogenase (SD),^78^ shikimate kinase (SK),^79^ and 5‐enoyl‐pyrovyl‐shikimate‐3‐phosphate synthase (EPSPS)^80^; in AROM, they are fused in the order _N_‐DHQS‐EPSPS‐SK‐DHQD‐SD‐_C_. Homodimerization of AROM was expected to be mediated by its DHQS domain, which is an obligate homodimeric enzyme, and possibly by its type I DHQD domain, which typically forms homodimers as well. Although the structures of the monofunctional homologs of the constituent domains are studied in great detail in prokaryotes and plants, the fungal AROM complex has so far withstood structural analysis at molecular detail.

**Figure 8:**
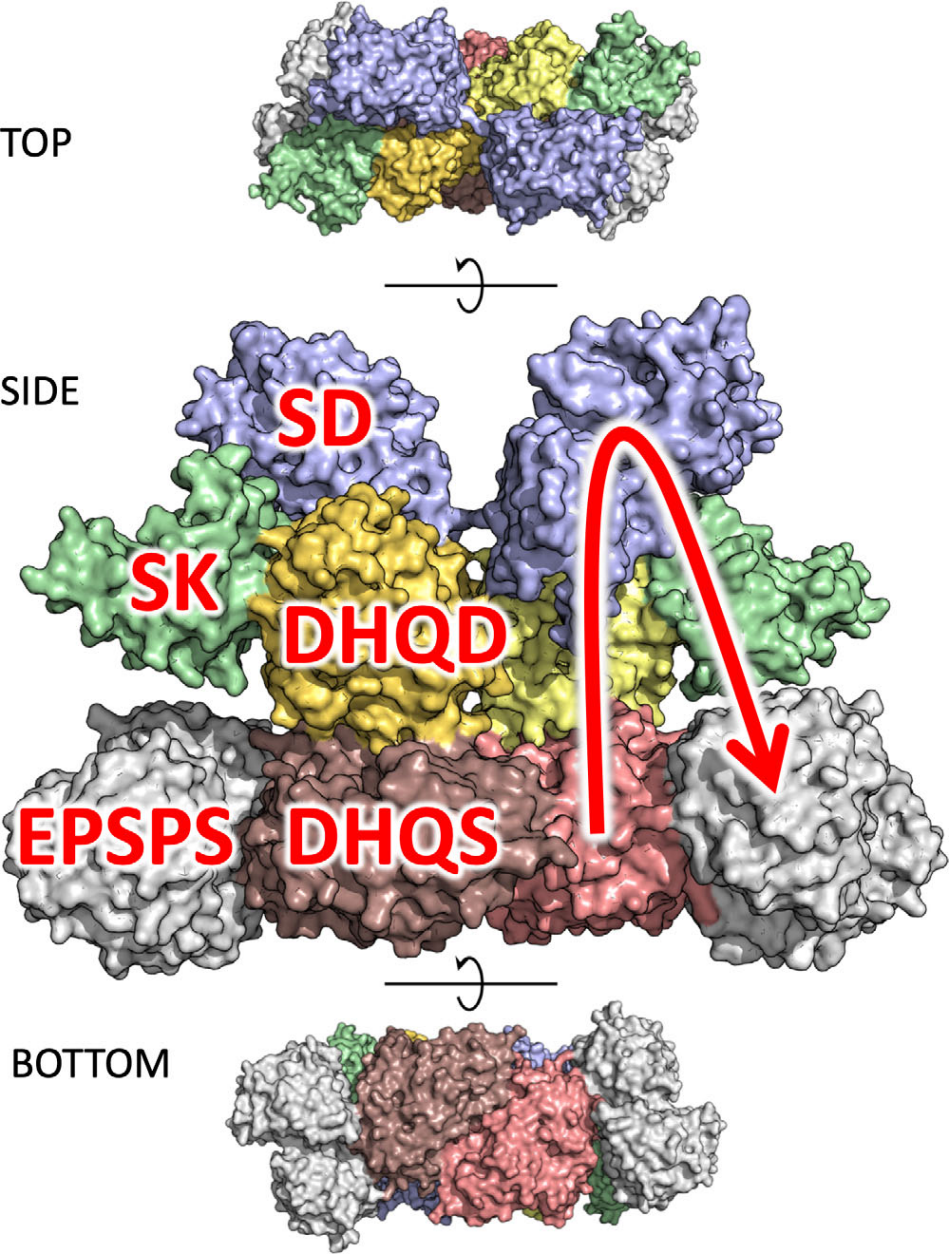
Crystal structure of the dimeric AROM complex in top, side (enlarged), and bottom view, with the five constituent enzymatic domains colored individually. In the side view, the domains are labeled on the left half of the dimer, on the right half the succession of metabolic steps is illustrated by an arrow, indicating the path DHQS‐DHQD‐SD‐SK‐EPSPS

We had recently set out for a new approach to study AROM's structure and function, making use of the thermophilic eukaryotic model organism *Chaetomium thermophilum* (*Ct*). *Ct*AROM (UniProt: G0S061) turned out to be a well‐behaved and stable homodimer of about 340 kDa (Table 1, Target: T0999). As we did not expect a fast breakthrough in crystallization experiments, we started to equip ourselves with experimental restraints for an in silico structure modeling. To this aim, we collected small angle X‐ray scattering (SAXS) data and, in collaboration with Alexander Leitner from ETH Zürich, cross‐linking mass‐spectrometry (XL‐MS) data, which we aimed to combine for a rigid‐body modeling and refinement approach based on the known structures of the individual enzymatic domains (see also^81^). However, we indeed obtained well‐diffracting crystals quite early on and thus focused on crystallographic structure solution. Finally, the crystal structure revealed a very compact assembly of the 10 domains, with the expected homodimeric DHQS and DHQD domains at the center (Figure 8). All individual domains exhibit the structure expected for their monofunctional counterparts, and the enzymatic domains catalyzing consecutive steps of the pathway are found in close proximity to each other.

Although not used for initial structure determination, the SAXS and XL‐MS data turned out to be very insightful in investigating the conformational landscape of the AROM complex (to be published), which motivated us to provide both our SAXS and XL‐MS data to CASP participants. Obviously, the prediction of the individual domains was a trivial task, and was mastered very well by most of the participating groups (average best GDT‐TS over D2‐D5 domains = 80.39). When it came to the prediction of the whole assembly, however, the situation was quite different. Although a number of predicted interdomain interfaces come close‐to what is observed in the crystal structure, it is hard to score the outcome in a quantitative fashion. For many predictions, individual pairs of domains that are consecutive in sequence are docked in a roughly correct orientation, but the transition from these roughly correct to clearly wrong orientations is smooth, and there is barely a prediction having three domains docked correctly per monomer. Notably, while the majority of the groups correctly constrained the DHQS domain to form the obligate homodimer, almost none of the participants put the respective constraint on the DHQD domain. We expect that this additional constraint would have helped in most cases to guide the assembly into the direction of the crystal structure. Overall, as no group could predict the AROM complex to an accuracy that reasonably reflects close‐to‐correct relative orientations of its constituent domains, we cannot clearly nominate the better predictions. It is noticeable, however, that the SAXS‐assisted predictions yielded the optically overall most‐native‐looking model, TS008_1 from the Pierce group, which also yielded the seventh best GDT‐TS score (22.9) and the third best global RMSD to the crystal structure: 27.9 å. A detailed description of data assisted modeling results is provided in a dedicated chapter of this special issue.

### 2.9 Apical end cap and needle of the antifeeding prophage (AFP) from *S. entomophila* and its threefold symmetric needle (CASP: H1021, PDB: 6RAP; CASP: H1022, PDB: 6RBK). Provided by Ambroise Desfosses, AK Mitra, and Irina Gutsche

Contractile injection systems (CISs) such as contractile bacteriophage tails, the Type VI secretion system (T6SS), R‐pyocins, and tailocins are multiprotein injection devices sharing a seringe‐like architecture.^82^, ^83^, ^84^ They are assembled of three major building blocks: a long rigid inner tube sharpened by a needle‐like tip, a contractile helical sheath surrounding the tube, and a baseplate that anchors the system to the target cell membrane, rearranges and triggers sheath contraction to expel the tube out of the sheath and puncture the target membrane. The two targets provided to CASP were derived from the high‐resolution cryo‐electron microscopy (cryo‐EM) structures of the antifeeding prophage AFP from the soil bacterium *S. entomophila* whose pathogenicity to the New Zealand pasture pest *Costelytra giveni*, is largely due to AFP which injects its insecticidal toxin into the *C. giveni* larvae.^85^ The targets represent the two opposite extremities of the AFP tailocin in its metastable extended state: the cap (Table 1, Target H1021) and the needle (Table 1, Target H1022).

**Figure 9:**
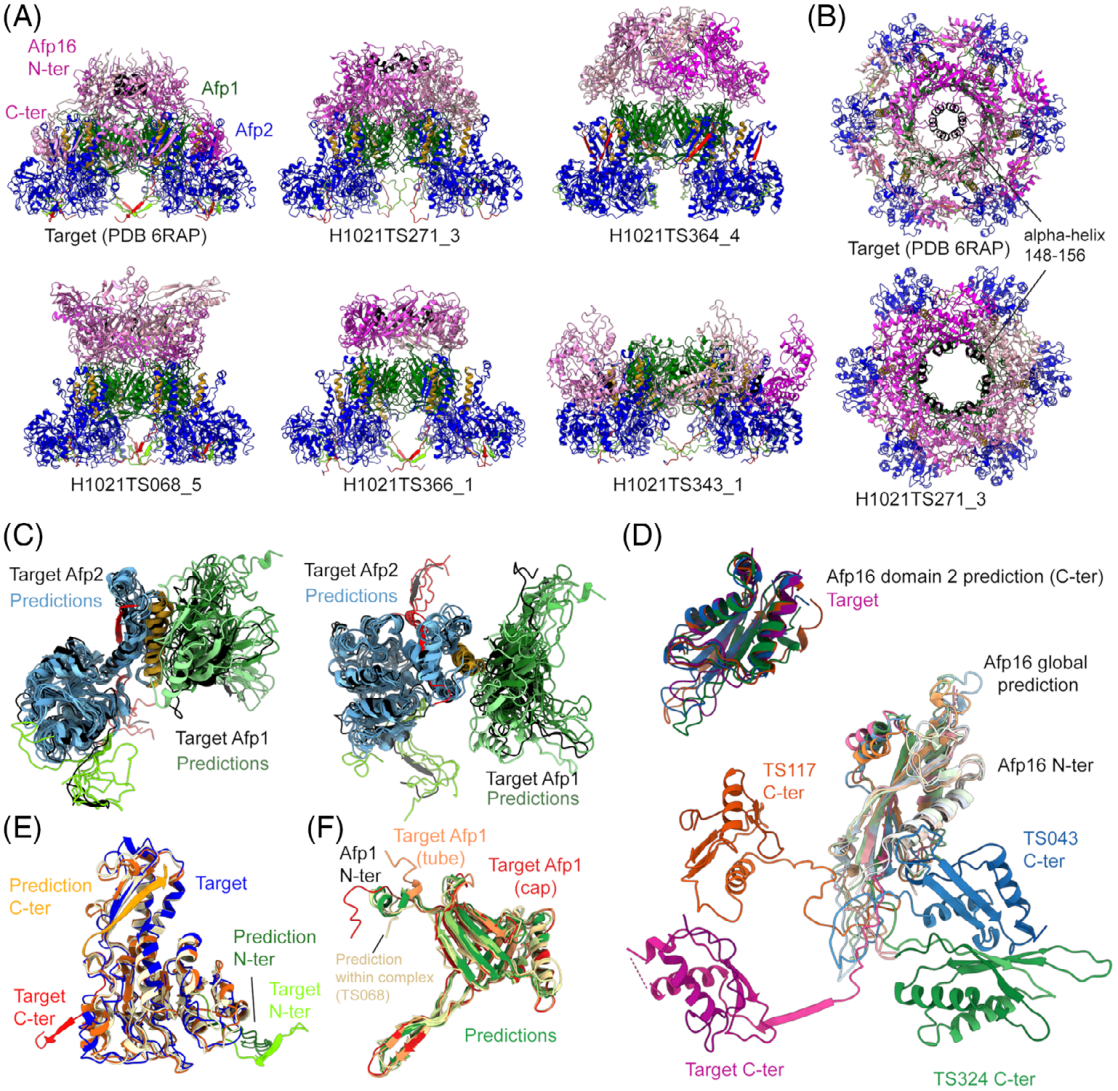
The apical cap of the AFP particle in extended state. A,B, Experimental structure of the H1021 target and the five best CASP prediction models according to QS score. This hexameric complex is composed of one layer of the sheath protein Afp2 (blue) surrounding the tube protein Afp1 (dark green) and capped by Afp16 (shades of magenta). The N‐ and C‐terminal arms of Afp2 are shown in light green and red, respectively. The α‐helix of Afp2 interacting with the tube is shown in orange. The ring‐forming α‐helix 148 to 156 is shown in black. C, Afp1‐Afp2 dimeric models are aligned to the target (black). D, Monomer predictions for Afp16, both for domain 2 only (up) and for the whole protein (bottom). EF, Monomer predictions for Afp2 and Afp1, respectively

The cryo‐EM map of the first target (H1021), determined at an average resolution of 3.3 å, corresponds to the sixfold symmetric apical end cap. This structure shows how the cap protein Afp16 (subunit 3 of H1021) binds to the inner tube protein Afp1 (subunit 1) and the sheath protein Afp2 (subunit 2) to tightly stabilize the tube and prevent toxin egress through the wrong extremity. Although homologous structures of the tube and sheath proteins in related injection systems such as the bacteriophage T4^86^ and the R‐pyocin^87^ had been solved, at the time of the CASP competition the cap protein Afp16 had no known structural homolog for the entire sequence length, and therefore represented a potentially difficult target for ab initio modeling. Accordingly, the predicted models of the Afp1‐Afp2‐Afp16 complex show a good accuracy for the Afp1‐Afp2 sub‐complex (Figure 9A,B). The conserved interface formed between the sheath and the tube (Afp2 α‐helix‐Afp1 ß‐sheets) was correctly modeled by the top predictions sorted by QS score (Figure 9C). The conserved bilobe fold of Afp2, described for other CIS sheath proteins, was accurately modeled in the monomer predictions (Figure 9E). In the complex, intra‐Afp2 interactions via the extended N‐ and C‐terminal arms are present in three out of five top models, although the monomer predictions for Afp2 showed a C‐terminal extension folded back onto the upper lobe of Afp2 (Figure 9E).

The tube proteins Afp1 has a similar fold as the T4 (gp19,^67^) the T6SS Hcp,^88^ and the R‐pyocin tube protein,^87^ predicted with high accuracy at the monomer level with GDT‐TS > 70 (Figure 9F). At the apical end, in the cap‐bound state, the N‐terminal Afp1 α‐helix unfolds to accommodate the interaction with Afp16 and to follow the Afp16 interdomain linker (Figure 9F). Interestingly, although the predicted models of the Afp1 monomer show an N‐terminal α‐helix as observed in related tube proteins and in the tube conformation of Afp1, several models of the whole complex presented a partially unfolded helix as in the cap conformation (Figure 9F, TS068).

In the complex, the position and interaction surfaces of the cap protein Afp16 were not precisely determined by the CASP prediction models (Figure 9A), likely due to the modular organization of this protein. Afp16 is composed of two distinct domains linked by a long loop, and forms a hexameric ring at the apex of the CIS trunk. The N‐terminal domain constitutes an extra layer of the tube while the C‐terminal domain caps the sheath (Figure 9A). CASP prediction models for Afp16 monomers show good accuracy for the N‐ and C‐terminal domains individually, but failed to predict their relative position (Figure 9D). The N‐terminal domain of Afp16 possesses a short α‐helix (148‐156), which organizes in a tight ring of 9 å in diameter that constricts the extremity of the particle upon Afp16 hexamerisation (Figure 9B). While several models correctly placed this helix toward the middle of the complex, the predicted interaction was less tight than observed in the target (Figure 9B).

**Figure 10:**
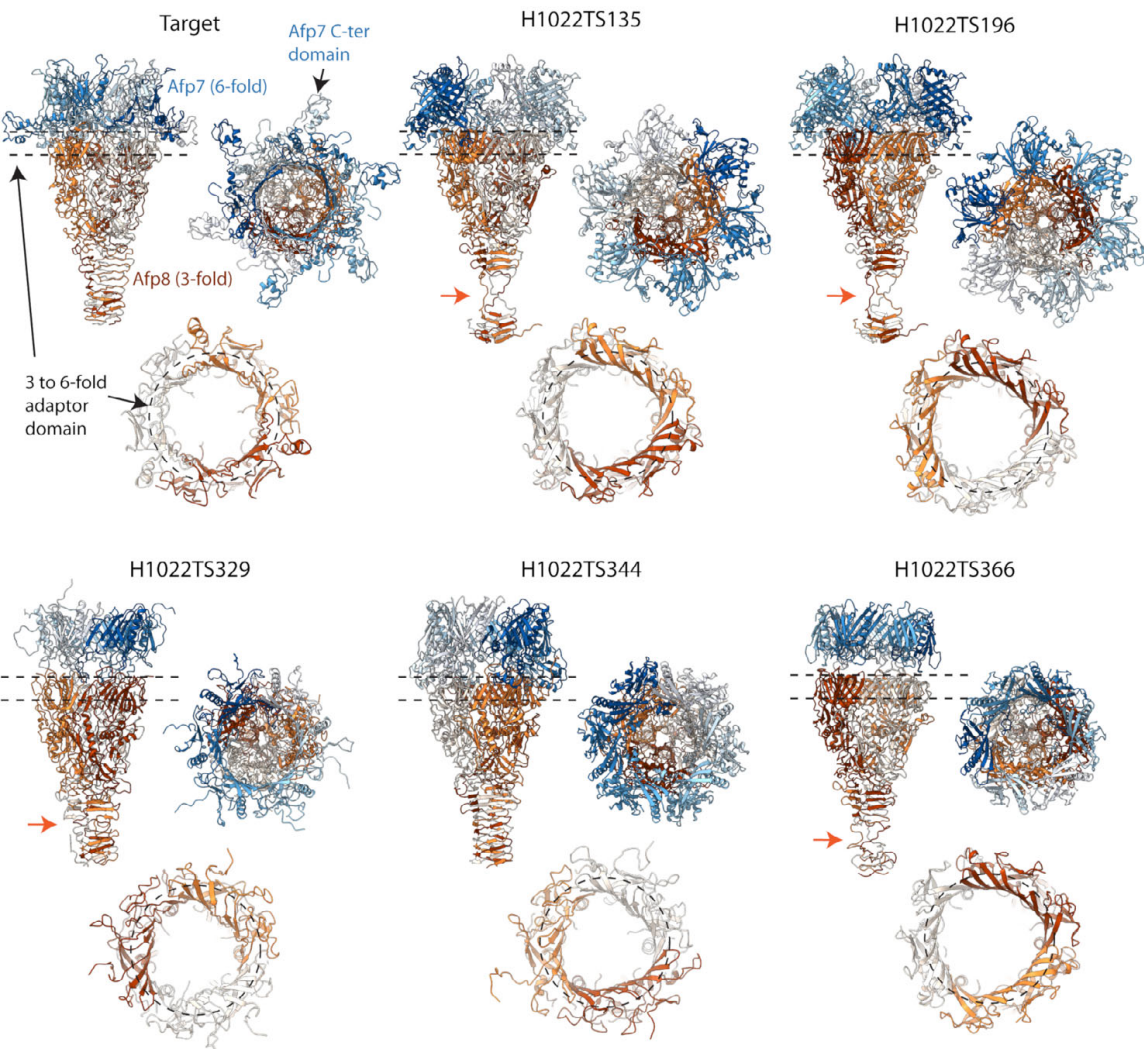
The needle of the AFP particle. Experimental structure of the target H1022 and the five best CASP prediction models according to QS score are shown in side view (top left), top view (top right), and as a slice through the region indicated by dashed lines in upper panel, corresponding to the threefold to sixfold adaptor domain of Afp8 (bottom, dashed circle). The six copies of Afp7 are shown as shades of blue, while the three copies of Afp8 forming the needle are shown in shades of orange. Orange arrows indicate the disruption of the ß‐helical wall observed in several CASP prediction models

The second target (H1022) corresponds to the threefold symmetric structure of the AFP needle. The original cryo‐EM map encompassing three copies of the needle protein Afp8 (Figure 10, shades of orange) and six copies of the tube initiator Afp7 (Figure 10, shades of blue), was solved to an average resolution of 3.4 å. Reminiscent of T4 and T6SS, an important feature of the needle hub are the twin ß‐barrel domains of Afp8 which have a fold similar to the core domain of the tube proteins (Afp1, Afp7 N‐terminal domain). Their arrangement creates a pseudohexameric layout^67^, ^89^ which, accommodates the transition from the hexameric ring formed by the tube initiator Afp7 to the C3‐symmetry of the rest of the Afp8 needle trimer (Figure 10, dashed circles). In the predicted models, the hexameric nature of this transition is not well respected, showing rather a triangular organization. Another important feature of this target is the tapered needle at the bottom of Afp8 with an intercalated ß‐helical wall analogous to the T4 gp5^89^ and T6SS VgrG/VgrG1,^82^ but notably shorter. This feature was well reproduced by the CASP prediction models, although most of them showed a disruption of the ß‐helical wall toward the bottom of the needle (Figure 10, orange arrows). Globally, the conserved fold of Afp8 was accurately modeled in the predictions. The structure of Afp7 was well predicted for the core N‐terminal domain at the interface with Afp8, whereas the position and the fold of the C‐terminal domain could not be modeled correctly.

### 2.10 Structure of a glycoside hydrolase family 31 α‐xylosidase in complex with a cleaved xyloglucan oligosaccharide (CASP: T1009; PDB: 6DRU). Provided by Hongnan Cao, Jonathan D. Walton, and George N. Phillips, Jr

Xyloglucan is a major hemicellulose of plant cell walls.^90^, ^91^ Cellulose and hemicellulose represent valuable biomass resources for renewable lignocellulosic biofuel production.^92^ A bottleneck to this process, that is, biomass recalcitrance, is the natural resistance of plant cell walls to microbial and enzymatic deconstruction.^92^ To overcome this, various enzymatic cocktails have been developed by DOE funded Bioenergy Research Centers and others for efficient and eco‐friendly conversion of these naturally abundant polysaccharides into readily fermentable sugar units. Walton's group at the DOE Great Lakes Bioenergy Research Center (GLBRC) have previously demonstrated that α‐xylosidase from *Aspergillus niger* (AxlA) enhances yields of monomeric sugars glucose and xylose from xyloglucan and hold promise in industrial lignocellulose conversion.^90^, ^91^ In order to elucidate the molecular basis of its catalytic function and substrate specificity, we determined the crystal structure of recombinant AxlA in complex with its catalytic product (Table 1, Target T1009; PDB: 6DRU), a xyloglucan fragment at 2.7 å resolution via molecular replacement using a structure of an α‐glucosidase (PDB: 4B9Y)^93^ in the same glycoside hydrolase family 31 (GH31, https://www.cazy.org 94) as AxlA.

**Figure 11:**
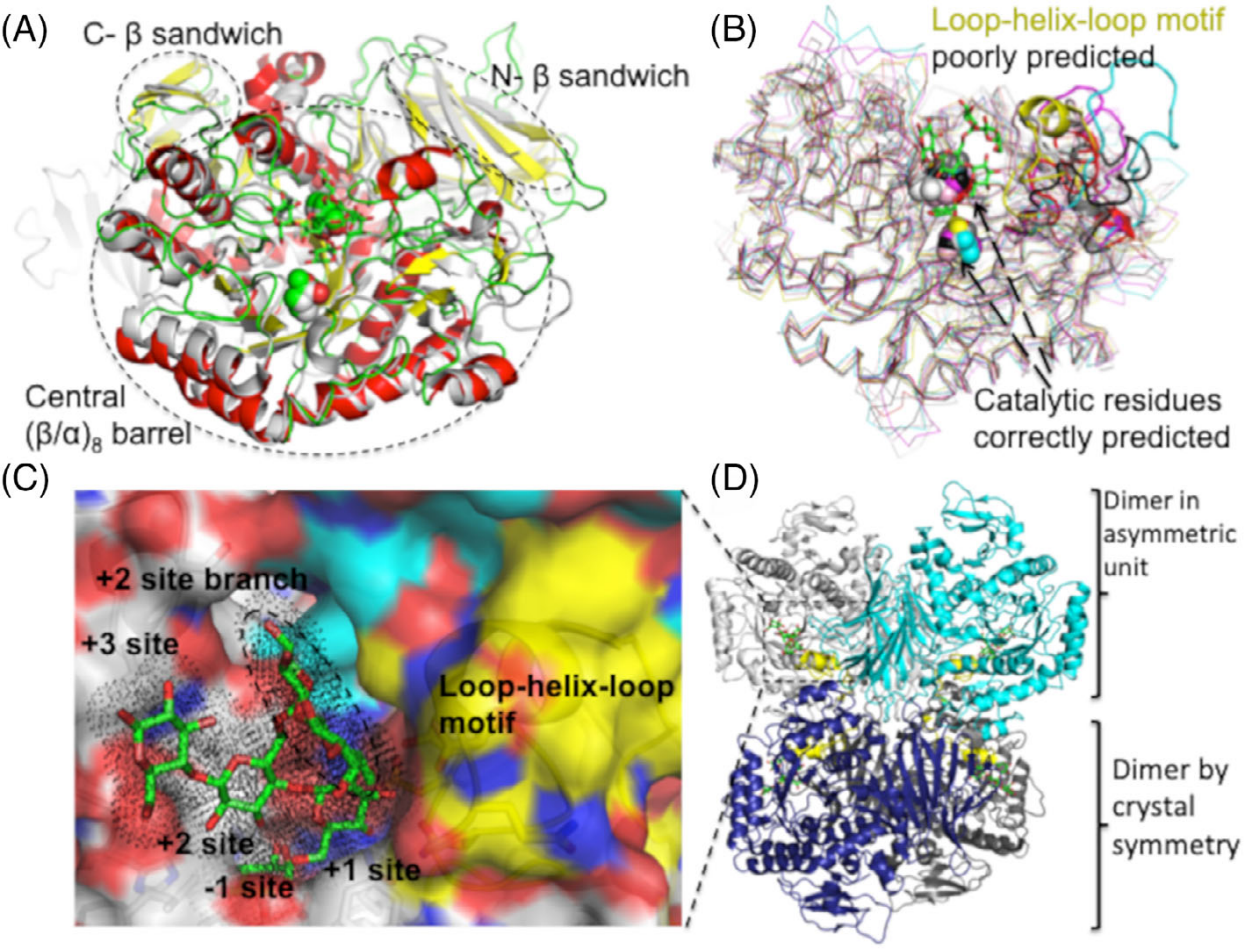
A Experimental structure of the target H1022 and the five best CASP prediction models by QS score (one per participating group) are shown in side view (top left), top view (top right), and as a slice through the region indicated by dashed lines in upper panel, corresponding to the threefold to sixfold adaptor domain of Afp8 (bottom, dashed circle). The 6 copies of Afp7 are shown as shades of blue, while the 3 copies of Afp8 forming the needle are shown in shades of orange.) Crystal structure of AxlA (PDB: 6DRU, chain A, color‐coded by secondary structures, helices in red, beta‐strands in yellow, and loops in green) and superposition with the search model (PDB: 4B9Y, white) shows conserved GH31 fold and putative catalytic residues (spheres). The cleaved xyloglucan oligosaccharide is shown as sticks with carbon in green and oxygen in red. B, Structure superposition of AxlA with top CASP13 regular target predictions shows an overall agreement on fold (thin ribbons) and conserved catalytic residues positions (spheres). A less conserved loop‐helix‐loop motif (cartoons) is poorly predicted and lacks agreement. Top CASP13 models are color‐coded as cyan (MUFold), pink (Jones‐UCL), magenta (wfAll‐Cheng), and Zhang (red) in the order ranked by GDT‐TS. Additional aligned models are in white (A7D), black (MULTICOM) and gray (QUARK), which are generated from top‐ranked prediction methods based on overall performance on all regular targets but outrun by others in AxlA predictions. C, A close‐up view in surface representation of AxlA substrate binding pocket with bound oligosaccharide product shown as sticks. Black dot surfaces indicate shape and interaction complementarity between the enzyme and the ligand. The ligand +1 site and branched sugar units attached to the +2 site are recognized by a pocket formed between the loop‐helix‐loop motif in yellow and an adjacent subunit in cyan. The catalytic labile α‐glycosydic bond is between the −1 site and + 1 site sugars. D, Tetrameric AxlA assembly predicted by EPPIC

The structure shows an overall typical GH31 enzyme fold with two structurally conserved putative catalytic residues residing in a central (β/α)_8_ barrel catalytic domain flanked by two β sandwich domains at N‐ and C‐termini (Figure 11A). The putative nucleophile and general acid residues Asp395 and Asp487 locate on the opposite sides of the catalytic labile C1 position of the xylose at −1 site cleaved from the branched xyloglucan fragment. The averaged distances between the carboxylic oxygen atoms are 6.38 and 6.55 å for the two structurally similar copies of the molecules in the asymmetric unit, respectively. These values are consistent with a retaining catalytic mechanism involving double displacement steps.^95^

The amino acid sequence of AxlA (UniProt ID: G3XMN9), without the N‐terminal peptide 5′‐MYFSSFLALGALVQAAAA‐3′, was submitted to CASP13 for both regular target and oligomeric structural predictions. The highest sequence identity of existing homologs to AxlA in PDB is ~28%. The top 4 ranked predicted models of AxlA as a regular target are from prediction participants MUFold (GDT‐TS = 71.24), Jones‐UCL (GDT‐TS = 71.20), wfAll‐Cheng (GDT‐TS = 69.08), and Zhang (GDT‐TS = 68.38). To our surprise, most overall top‐ranked teams like AlphaFold A7D (GDT‐TS = 60.97), QUARK (GDT‐TS = 64.10), and MULTICOM (GDT‐TS = 67.13) are out of top 10 in this particular case, except Zhang. We also noticed that the overall fold and locations of the two proposed catalytic residues of AxlA is successfully predicted by most top ranked models. However, not a single predicted model is able to reproduce the experimentally determined loop‐helix‐loop motif structure (residues 398‐425), which appears to be structurally essential for AxlA to form +1 site for the backbone glucose as well as recognizing galactose and xylose sugars on the adjacent branch of the xyloglucan fragment being acted on (Figure 11B,C). This is possibly due to the low sequence and structural conservation in this region in structurally characterized GH31 enzymes, which comprise a wide variety of substrate specificities according to https://www.cazy.org,^94^ including α‐glucosidase (EC 3.2.1.20); α‐galactosidase (EC 3.2.1.22); α‐mannosidase (EC 3.2.1.24); α‐1,3‐glucosidase (EC 3.2.1.84); sucrase‐isomaltase (EC 3.2.1.48) (EC 3.2.1.10); α‐xylosidase (EC 3.2.1.177); α‐glucan lyase (EC 4.2.2.13); isomaltosyltransferase (EC 2.4.1.‐); α‐1,4‐glucosyltransferase (EC 2.4.1.161); sulfoquinovosidase (EC 3.2.1.‐). The shape and interactions complementarity between the enzyme pocket and the xyloglucan fragment provides further evidence on its substrate specificity and suggests its usefulness in lignocelluloses deconstruction (Figure 11C).

A close examination on the quaternary assembly of AxlA using EPPIC^96^ identified it as a possible homo‐tetramer formed from two subunits in the asymmetric unit and two adjacent subunits related by crystallographic symmetry. The above‐mentioned loop region with poor structural predictions is also involved in tetrameric assembly (Figure 11D). This can be one of the factors that lead to the relatively poor predictions of AxlA as oligomeric target, with the top ranked model from BAKER team giving a QS‐score of only 0.177 for the dimer interface.

### 2.11 Structure of *A. thaliana* xylan *O*‐acetyltransferase 1 (CASP: T0969, PDB: 6CCI). Provided by Markus Alahuhta, Vladimir V. Lunin, and Yannick J. Bomble

*A. thaliana* xylan *O*‐acetyltransferase 1 (AtXOAT1) is a plant‐specific trichome birefringence (TBL) like enzyme that catalyzes the 2‐*O*‐acetylation of the xylan backbone.^97^ Structural characterization of AtXIAT1 is part of a wider effort to elucidate the molecular basis of polysaccharide biosynthesis and biomass modifications by different plant‐derived enzymes, including glycosyltransferases, acetyltransferases, and methyltransferases.

**Figure 12:**
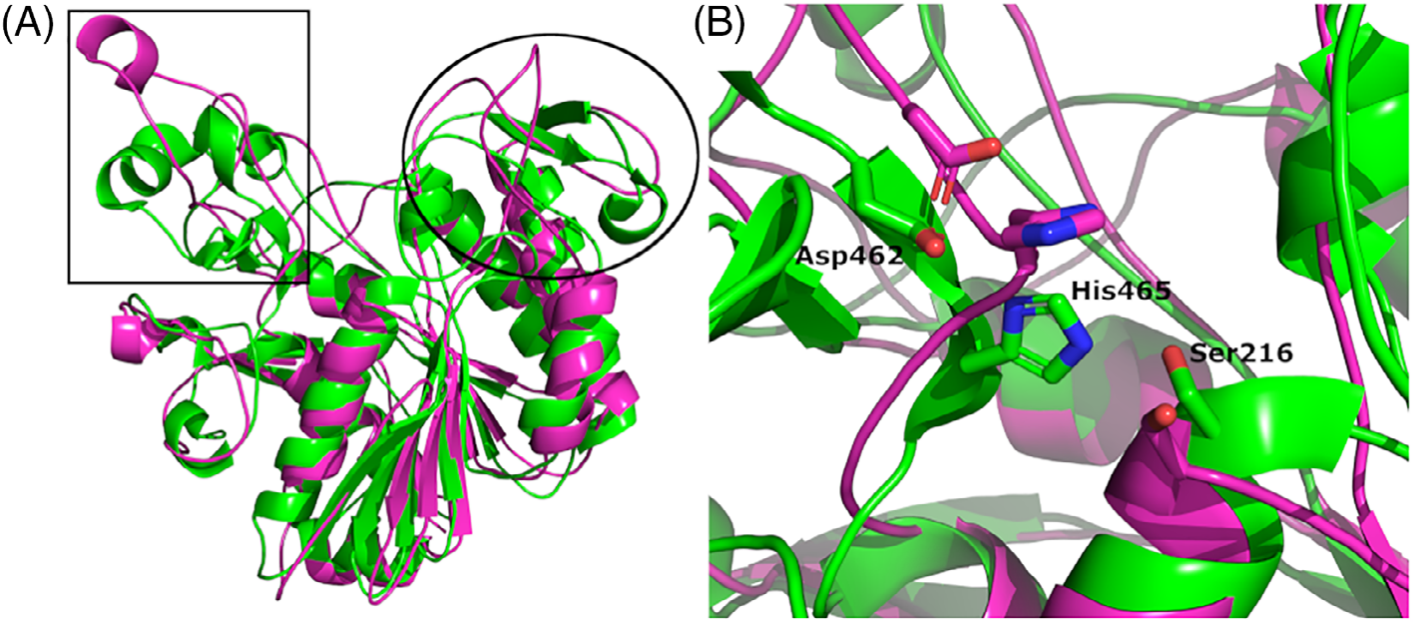
A, Superposition of the *At*XOAT1 crystal structure (green) and top CASP13 model T0969TS197_1‐D1 (magenta). B, A close‐up view of the active site showing the catalytic triad in stick representation

The crystal structure of the *At*XOAT1 was determined by single wavelength anomalous dispersion method using anomalous signal from sulfur atoms (S‐SAD) at CuK_α_ wavelength (Table 1, Target: T0969). The structure is composed of three β‐sheets consisting of seven (β3‐β6, β9, β12‐β13), four (β7‐β8, β10, β11), and two (β1‐β2) β‐strands, respectively, nine α‐helices including a “broken” one and three α‐helical turns (Figure 12A). The molecule is divided into two lobes with a deep cleft in between: the first larger lobe contains almost all secondary structure elements found in the protein while the second, of smaller size, is mostly unstructured and contains four disulfide bridges (Cys140‐Cys191, Cys162‐Cys227, Cys171‐Cys467, Cys384‐Cys463). The deep cleft between the two lobes contains the substrate binding site, that is, a catalytic triad Ser216‐His465‐Asp462 similar to that found in serine proteases^98^ (Figure 12B). The walls of the cleft are formed by two flexible loops (residues 443‐448 and 273‐281). Biochemical experiments with truncation mutants at the N‐terminal cytoplasmic tail, the predicted transmembrane domain and the N‐terminal variable region, identified the catalytic domain as the region between residues 133 to 478.

The top two ranked models (T0969TS197_1‐D1 and T0969TS406_1‐D1, from MESHI and Seder3mm groups, GDT‐TS = 58.19 and 57.49, respectively) correctly reproduced the overall fold of *At*XOAT1 (Figure 12A). Most deviations from the reference structure (PDB: 6CCI) occur at the region surrounding residues 437 to 468 and at loops 262 to 284 and 312 to 334. The short β‐strands defined by residues 437 to 439 and 462 to 464 are also not reproduced in the models. A closer inspection of the active site revealed an incorrect orientation of two of the three catalytic residues, namely Asp462 and His465, with a main‐chain shift toward the top of the cleft and overall RMSD of the active site residues of 3.7 å and 4 å, respectively. This is accompanied by an incorrect flip of residues Trp309 and Trp466 into the active site.

The third ranked model T0969TS274_1‐D1 (MUFold) showed a slightly higher global deviation from the reference structure (GDT‐TS = 52.83) but still captured the correct overall fold. Interestingly, this model shows a slightly better local accuracy, with an RMSD of the active site residues of 3.2 å (Figure 12B). In summary, the top three models all reproduced the overall fold and most of the secondary structure elements. They all had shifts in the position of the catalytic residues but were close enough to be able to correctly identify the location of the active site and identity of catalytic residues.

## 3 CONCLUSIONS

This article provides insights into structural and functional details of 13 selected CASP13 targets and analyses to what extent the most interesting features of the targets are reproduced in the models, as illustrated by the author of the structures. The presented examples highlight a series of recurring themes, reflecting both the success and pitfalls of current prediction methods. On one hand, the ability of predicting hard protein folds at the tertiary level has increased enormously with the structure of many difficult targets reproduced with impressive accuracy. On the other hand, important global and local features of prediction models are still seldom as accurate as in the experimental structure. This is the case of enzyme active sites and ligand binding sites, where the predicted arrangement of the amino acids side chains involved in ligand binding and substrate specificity has not achieved the level of accuracy required to confidently infer their function (i.e., T0961, T0957, T0969). Accurate prediction of loops is still a challenging task. As they are often involved in PPIs, their incorrect prediction can compromise the accuracy of the interacting surface and overall structure of the complex. An example of this scenario is highlighted in T1009, where a specific loop‐helix‐loop motif appears to be essential for both catalytic activity and tetrameric assembly of AxlA. A similar case is target H1021, where the incorrect prediction of a long loop region leads to inaccurate domain orientation of the Afp16 subunits. The latter example also points to an important, re‐emerging issue in CASP. That is, the ability of current methods in modeling the correct quaternary structure of proteins remains rudimentary and shows little progress compared to what observed at the tertiary level. For example, while the majority of predictors successfully modeled the individual domains of the AROM enzyme (T0999), no group was able to predict the correct assembly of the pentafunctional complex. This represents an important drawback, as the oligomeric state is often relevant for structural and functional integrity of the target and may also assist predictions of the interdomain orientation in multidomain proteins. As also highlighted in H0953, however, the oligomeric state of the target was possibly not taken into account for predictions.

As assessing the functional relevance of models is difficult for CASP assessors to address on a large scale, we hope that this study will inspire future CASP assessors in emphasizing the relevant aspects of models that inform our understanding of protein function.

## AUTHOR CONTRIBUTIONS

Concept, abstract, introduction, editing, and coordination––by R.L., A.K., K.F., J.M., T.S., and M.T.; target‐specific sections––by authors provided in the sections' titles.

## Supporting information


**TABLE S1** CASP13 target providers.
