# Supplementary material for: Target highlights in CASP13: Experimental target structures through the eyes of their authors
Source: Proteins. 2019 Sep 9;87(12):1037–57. doi: 10.1002/prot.25805 (PMC6851490; doi:10.1002/prot.25805)
Supplement: Supplementary file 1 — TABLE S1 CASP13 target providers. [file PROT-87-1037-s001.docx]

| # | Contibutor | Country | Institution | # | Contibutor | Country | Institution |
| --- | --- | --- | --- | --- | --- | --- | --- |
| 1 | Tom Peat | Australia | CSIRO | 19 | Xiaochen Bai | USA | UT SouthWest MC |
| 2 | Leila Lo Leggio | Denmark | U Kobenhavn | 20 | George Minasov | USA | NorthWestern U |
| 3 | Ambroise Desfosses | France | IBS, Grenoble | 21 | Lindsey Spiegelman | USA | UC San Diego |
| 4 | Marianne Ilbert | France | CNRS, Paris | 22 | Chi-Lin Tsai | USA | UTMD Anderson |
| 5 | Michael Groll | Germany | Tech U Munchen | 23 | Manal Swairjo | USA | San Diego State |
| 6 | Andrei Lupas, Marcus Hartmann | Germany | Max Planck Tübingen | 24 | Andrzej Joachimiak,  Karolina Michalska,  Kemin Tan | USA | Argonne Lab |
| 7 | Noa Keren | Israel | Ben Gurion U | 25 | Thomas Szyperski | USA | U Washington |
| 8 | Kaspars Tars | Latvia | BRCS, Riga | 26 | Gaetano Montelione | USA | U Rutgers |
| 9 | Shabir Najmudin | Portugal | U Lisbon | 27 | Garry Buchko | USA | Pacific NW Natl Lab |
| 10 | Stefan Arold | Saudi Arabia | KAUST | 28 | Petr Leiman | USA | U Texas MB |
| 11 | Mark van Raaij | Spain | CSIS, Madrid | 29 | Damian Ekiert | USA | New York U |
| 12 | Tilman Schirmer | Switzerland | U Basel | 30 | George Phillips, Jonathan Clinger,  Mitchell Miller | USA | U Rice |
| 13 | Brian Trevor Sewell | So. Africa | U Cape Town | 31 | Hong Zhou | USA | UC Los Angeles |
| 14 | Brian Marsden | UK /Canada | SGC, Oxford/Toronto | 32 | Hongnan Cao | USA | Georgia I Tech |
| 15 | Owen Davies | UK | U Newcastle | 33 | Henrique Pereira | USA | L Berkeley Lab |
| 16 | Arnaud Basle | UK | U Newcastle | 34 | Oliver Clarke | USA | Columbia U |
| 17 | Andrew Lovering | UK | U Birmingham | 35 | Phoebe Rice | USA | U Chicago |
| 18 | Adam Frost | USA | UC San Francisco | 36 | Marcus Alahuhta | USA | Natl Renewable Energy Lab |

**TABLE S1.** CASP13 target providers.
